# Impact of the ABO and RhD Blood Groups on the Evaluation of the Erythroprotective Potential of Fucoxanthin, β-Carotene, Gallic Acid, Quercetin and Ascorbic Acid as Therapeutic Agents against Oxidative Stress

**DOI:** 10.3390/antiox12122092

**Published:** 2023-12-08

**Authors:** Ricardo Iván González-Vega, Miguel Ángel Robles-García, Litzy Yadira Mendoza-Urizabel, Kelly Nabil Cárdenas-Enríquez, Saúl Ruiz-Cruz, Melesio Gutiérrez-Lomelí, Rey David Iturralde-García, María Guadalupe Avila-Novoa, Fridha Viridiana Villalpando-Vargas, Carmen Lizette Del-Toro-Sánchez

**Affiliations:** 1 Department of Medical and Life Sciences, Cienega University Center (CUCIÉNEGA), Universidad de Guadalajara, Av. Universidad 1115, Lindavista, Ocotlán 47820, Jalisco, Mexico; ricardo.gonzalez@academicos.udg.mx (R.I.G.-V.); melesio.gutierrez@academicos.udg.mx (M.G.-L.); maria.anovoa@academicos.udg.mx (M.G.A.-N.); 2 Department of Cellular and Molecular Biology, University Center for Biological and Agricultural Sciences (CUCBA), Universidad de Guadalajara, Periférico Norte N° 799 Núcleo Universitario, C. Prol. Belenes, Zapopan 45100, Jalisco, Mexico; viridiana.villalpando@academicos.udg.mx; 3 Department of Research and Postgraduate in Food, Universidad de Sonora, Blvd Luis Encinas y Rosales S/N, Col. Centro, Hermosillo 83000, Sonora, Mexico; a217204183@unison.mx (L.Y.M.-U.); knabilc12@gmail.com (K.N.C.-E.); saul.ruizcruz@unison.mx (S.R.-C.); rey.iturralde@unison.mx (R.D.I.-G.); 4 Department of Health Sciences, University Center of the Valleys (CUVALLE), Universidad de Guadalajara, Carr. a Guadalajara Km. 45.5, Ameca 46600, Jalisco, Mexico

**Keywords:** blood groups, antioxidant, erythroprotective potential, oxidative stress

## Abstract

Previous studies detail that different blood groups are associated with incidence of oxidative stress-related diseases such as certain carcinomas. Bioactive compounds represent an alternative for preventing this oxidative stress. The aim of this study was to elucidate the impact of blood groups on the erythroprotective potential of fucoxanthin, β-Carotene, gallic acid, quercetin and ascorbic acid as therapeutic agents against oxidative stress. The impact of ABO blood groups on the erythroprotective potential was evaluated via the antioxidant capacity, blood biocompatibility, blood susceptibility and erythroprotective potential (membrane stabilization, in vitro photostability and antihemolytic activity). All tested antioxidants exhibited a high antioxidant capacity and presented the ability to inhibit ROO•-induced oxidative stress without compromising the cell membrane, providing erythroprotective effects dependent on the blood group, effects that increased in the presence of antigen A. These results are very important, since it has been documented that antigen A is associated with breast and skin cancer. These results revealed a probable relationship between different erythrocyte antigens with erythroprotective potential, highlighting the importance of bio-targeted drugs for groups most susceptible to certain chronic-degenerative pathologies. These compounds could be applied as additive, nutraceutical or encapsulated to improve their bioaccessibility.

## 1. Introduction

Reactive oxygen species (ROS), such as superoxide anion (O_2_^•−^), hydroxyl radical (•OH) and peroxyl radical (ROO•), are highly reactive unstable by-products of cellular metabolism, originally produced in mitochondria [1]. Free radicals are generally produced through electron transfer reactions in cells with or without enzymatic intervention. These reactions can be mediated via metal ions in transition, e.g., the •OH radical is generated in the presence of H_2_O_2_ with copper (Cu^+2^) or Iron (Fe^+2^) ions [2]. In adequate concentrations, reactive oxygen species are essential for cellular and immunological functions [3]. The respiratory discharge of phagocytes activated by bacterial stimulation is directed to produce oxygen metabolites. These free radicals are intended to degrade phagocytosed parasitic bacteria [2]. The bactericidal capacity of free radicals is very efficient; nevertheless, a considerable increase can damage the bacteria, as well as the environment in which the phagocytes act, resulting in damage to the body’s cells. Uncontrolled respiratory discharge is associated with chronic inflammation that can stimulate neoplastic processes since free radicals accumulate in cells and damage molecules such as DNA, lipids and proteins [4].

The involvement of uncontrolled respiratory discharges stimulates the degradation of biomolecules; this action is associated with non-communicable diseases such as cancer. Chronic degenerative diseases caused 74% of deaths around the world in 2019, with more than 40,805 million deaths recorded. Therefore, the intervention of the health and research sector for the prevention of neoplasias associated with cellular oxidative stress is urgent. Antioxidants such as carotenoids, phenols, flavonoids, and vitamins represent an alternative for reducing oxidative stress [5]. These biomolecules maintain a low redox state in cells through mechanisms of direct action on reactive species. The free radical scavenging capacity of antioxidants is related to Sequential Proton Loss Electron Transfer (SPLET), Single Electron Transfer (SET) and Hydrogen Atom Transfer (HAT) mechanisms [5,6]. The antiradical activity of carotenoids is mainly due to the highly unsaturated chromophore and the functional groups in the case of xanthophylls. β-Carotene specifically presents an SET mechanism, due to its chemical nature being incapable of donating protons. However, fucoxanthin has the ability to transfer both hydrogen atoms and electrons through its double bonds and the -OH and epoxy functional groups. The free radical scavenging process of phenols and flavonoids is linked to successive deprotonations in their aromatic rings [6,7]. For some phenolic acids, the thermodynamically preferred deprotonation sites are the 4′-OH, 5-OH and 7-OH groups, a mechanism presented by SPLET compared to HAT or SET [6,8]. Vitamin C (L-ascorbic acid) is an important reducing agent, capable of donating electrons and protons to receptor molecules such as free radicals. In relation to the reducing potentials of antioxidants, they play a very important role in inhibiting oxidation and cytoprotective effects (neuroprotective, hepatoprotective and erythroprotective effects), preventing the development of chronic diseases related to oxidative stress, such as neurological [9], hemolytic [10,11,12] and carcinogenic processes [11,13].

Some carcinogenic processes are associated with different ABO phenotypes’ presence in the cell and tissues. Recent epidemiological research has reported the association between the risk of contracting non-communicable and communicable chronic diseases with the different ABO and Rh blood groups of human erythrocytes. Blood group A is associated with the incidence of different types of cancer (breast, gastrointestinal, liver, lung, pancreas, stomach, and non-melanoma skin cancer) and virally transmitted diseases (COVID-19) [14]. In our research, blood susceptibility studies were carried out on ultraviolet radiation-induced oxidative stress, in order to promote research that will use ABO blood groups as a descriptive factor to categorize patients with skin-related pathologies. It is expected that the results of the study will help us understand the possible effect of ultraviolet radiation’s (UVR’s) metastatic potential, pathological development and prevention in subjects at higher risk of developing non-melanoma skin cancer associated with blood groups [15]. It is known that UVR causes damage to genetic material, classifying it as an important factor for the development of skin cancer, mainly non-melanoma, and squamous cell carcinoma of the skin. Previously, a possible association between blood groups and the risk of skin cancer induced by prolonged exposure to ultraviolet radiation was demonstrated [15]. To date, studies suggest focusing on finding photoprotective compounds for groups A RhD-ve, AB RhD+ve and O (RhD not specified) that inhibit oxidative damage induced by UV radiation, since a higher incidence of cancer has been reported in these blood groups [15]. UVR induces peroxyl and alkoxyl radicals that react with the lipids and proteins of the erythrocyte membrane. Respiratory discharge AAPH [2,2′-azinobis (3-ethylbenzothiazolin)-6-sulfonic acid]-free radical initiator stimulation could be the key to elucidating the blood susceptibility of different blood phenotypes to chemical oxidation [16].

Recent research suggests that microalgae extracts act as important erythroprotective agents, reducing oxidative stress depending on the surface antigen of the erythrocytes [10,11,12]. Fucoxanthin and β-Carotene are among the most important and abundant marine carotenoids in algae and microalgae since they contribute more than 10% of the total estimated production of carotenoids in the marine environment [17]. These pigments have generated great interest in the pharmaceutical food industry due to their various health-beneficial properties, promoting pharmacological effects related to oxidative stress [13,18,19]. Previous studies have shown that phenolic extracts from beans [20], chickpea [21] and sorghum [22] present high antioxidant activity with erythroprotective properties [9]. However, the impact of ABO and RhD blood groups on the evaluation of the erythroprotective potential of fucoxanthin, β-Carotene, gallic acid, quercetin and ascorbic acid as therapeutic agents against oxidative stress has not been reported. The use of human erythrocytes as a cellular model of ABO antigens could contribute to the development of functional foods designed for people with a specific blood type, preventing chronic-degenerative diseases that affect a more susceptible group.

## 2. Materials and Methods

### 2.1. Reagents

All chemical reagents such as fucoxanthin (FXN), β-Carotene (β-Car), gallic acid (GA), quercetin (QUE), ascorbic acid (AA), dimethyl sulfoxide (DMSO), DPPH (1,1-diphenyl-2-picrylhydrazyl), ABTS [2,2′-azinobis (3-ethylbenzothiazolin)-6-sulfonic acid], sodium acetate buffer, FeCl3 (ferric chloride), TPTZ (2,4,6-tripyridyl-s-triazine), hydrochloric acid (HCl), Triton X-100, AAPH [2,2′-Azobis (2-methylpropionamidine) dihydrochloride] and PBS (phosphate buffer saline) were purchased from Sigma-Aldrich Co. (San Luis, MO, USA). All other chemicals and solvents used for this study are of the highest commercial quality.

### 2.2. Biological Material

All methodologies that employed human red blood cells (RBCs) were performed following the Mexican (NOM-253-SSA1-2012) and international (FDA: CFR—Code of Federal Regulations Title 21, part 640, Additional Standards for human blood and blood product, Support. B Red blood cells, Sec. 640.14, Testing the blood (21CFR640.14)) regulations. The membranes of RBCs with different ABO and RhD blood types were generously donated by the clinical analysis laboratory of the University of Guadalajara. The clinical laboratory is accredited by ISO-IEC 17,025 (NMX-EC-17025) [23] and ISO 15,189 [24], elaborated by technical committee ISO/TC 212 [25] (Clinical Laboratory Testing and In vitro Diagnostic Systems), taking as reference ISO/IEC 17,025 and ISO 9001 [26] [Mexican Standard: General requirements for the competence of testing and calibration laboratories. Institute for Standardization and Certification, A. C. (IMNC). Mexican Standard PROY-NMX-EC17,025 prepared by the Technical Committee for National Standardization of the Quality System INMC/CTNN 9, 2022]. The human erythrocytes were collected from healthy adult volunteers (25 to 40 years old) that contained approximately 4.7 to 6.1 × 10^6^ cells/μL. Previously, the information for the procedure had been provided, obtaining each one’s informed consent. The venipuncture technique was applied to collect human RBCs using a sterile vial with anticoagulant (EDTA). For these studies, the RBC samples were processed immediately after extraction for further experimental analysis. The purpose of these studies is to use RBCs as a membrane model to assess the impact of the ABO and RhD blood groups on the erythroprotective potential of FXN; therefore, the object of study does not focus on volunteers.

### 2.3. Preparation of Samples

FXN, β-Car, GA, QUE and AA were dissolved previously in DMSO. Once the samples were solubilized in DMSO, aliquots were taken to prepare the samples at different concentrations (0.5–10 µM) in a DMSO/PBS (1:9) solution. The purpose of preparing the solutions in DMSO/PBS is to prevent erythrocyte hemolysis [27]. After a detailed search, the literature reports that these compound concentrations in blood plasma range from 0.1 to 0.9 µM.

### 2.4. Determination of Antioxidant Capacity

The antioxidant capacity was determined in FXN, β-Car, GA, QUE and AA using three recognized methods. A 2,2-diphenyl-1-picrylhydrazyl (DPPH•) radical scavenging assay was performed, following the procedure outlined by Brand-Williams et al. [28]. A 2,2′-azino-bis(3-ethylbenzothiazoline-6-sulfonic acid) (ABTS•+) radical scavenging assay was conducted according to the method described by Re et al. [29] A ferric reducing antioxidant power (FRAP) assay was carried out as reported by Benzie and Strain [30]. The results were expressed as μmol TE (Trolox equivalents)/g for each antioxidant compound.

### 2.5. Erythroprotective Potential

#### 2.5.1. Blood Biocompatibility

The blood biocompatibility of FXN, β-Car, GA, QUE and AA was confirmed via direct hemolysis assay [11] on erythrocytes of the ABO and RhD blood system. RBCs samples were collected via venipuncture in EDTA tubes as described in the biological material section. A suspension of 10% erythrocytes was prepared through being washed three times with PBS (phosphate-buffered saline; 0.15 M; pH 7.4), removing the total plasma by centrifugation (2000× *g* for 10 min) and recovering the globular package. A quantity of 100 μL of erythrocytes suspension (10%) + 150 μL of each of the samples (*v*/*v*) at different concentrations (0.5 to 100 µM) + 100 μL of PBS was incubated at 37 °C. The erythrocytes were exposed for 6 h to the samples to evaluate direct hemolysis, measured at 2 h intervals. After concluding the incubation, each sample was centrifuged after adding 1 mL of PBS. A 300 µL volume of supernatant was measured at 540 nm in a 96-well microplate reader per triplicate. PBS buffer and Triton X-100 1% were used as negative and positive controls, respectively. Results were expressed as percentages of hemolysis and calculated using the following Equation (1):

(1) $$\%\;of\;hemolysis=\frac{A_{sample}-A_{PBS}}{A_{Triton}-A_{PBS}}\times 100$$

#### 2.5.2. Membrane Stabilization Assay

Heat-induced hemolysis and hypotonicity-induced hemolysis assays were used to evaluate the membrane stabilization (%) of all tested compounds, and diclofenac sodium (DS) was a standard drug on erythrocyte membranes with different antigen types. Diclofenac sodium (DS) was used as a control, since it is an anti-inflammatory commonly used as an analgesic and antipyretic in the treatment of acute rheumatic diseases and rheumatoid arthritis, decreasing the inflammatory response by inhibiting the release of lysosomal constituents from activated neutrophils, action that is carried out by stabilizing the lysosomal membrane. Therefore, using DS as a control in the Membrane-Stabilizing Capacity Assay is important for elucidating the potential of antioxidants as erythroprotective compounds. On the other hand, diclofenac sodium has adverse health effects. The erythroprotective antioxidants used in our study could act as novel and natural anti-inflammatory compounds [12].

A heat-induced hemolysis assay was carried out according to the methodology by Agarwal et al. [31] with slight modification. The reaction mixture containing 150 μL of erythrocytes suspension (2%) + 150 μL of each sample (10 µM) was incubated in a water bath at 55 °C for 30 min. After the time of incubation, 1 mL of PBS was added to each sample vial and centrifuged at 2000× *g* for 10 min. Subsequently, the supernatant was recovered, where 300 µL was taken to a microplate of 96 wells and read at an absorbance of 540 nm. An amount of 300 µL of erythrocytes suspension was used as a positive control in the same controlled condition. The membrane stabilization (%) was reported via the following Equation (2):

(2) $$\%\;of\;Membrane\;stabilization=\frac{A_{Control}-{(A}_{Sample}-A_{PBS})}{A_{Control}}\times 100$$

For the hypotonicity-induced hemolysis assay, the methodology of Agarwal et al. [31] was employed with some modification. Distilled water was used as a hypotonic solution since it has a lower concentration of solutes. Distilled water induces a lower osmotic pressure compared to other solutions. In erythrocytes, this solution would be intracellular fluid. The reaction mixture was carried out using 50 μL of RBC suspension (2%) + 100 μL of PBS + 100 μL of each sample (10 µM) + 200 μL of hyposaline solution in a water bath at 37 °C for 30 min. After incubation was complete, 850 μL of PBS was added to each sample vial and centrifuged at 2000× *g* for 10 min. As a negative control, we used 50 μL of RBC suspension (2%) + 400 μL PBS, and as a positive control, we used 50 μL of RBC suspension (2%) + 200 μL PBS + 200 μL hyposaline solution. The membrane stabilization (%) was calculated using Equation (2).

#### 2.5.3. Blood Susceptibility Test against Oxidative Stress

Blood susceptibility was determined on all erythrocyte blood phenotypes. The cell oxidative damage was induced via ultraviolet radiation (UVR) and chemically using the free radical generator AAPH [2,2′-Azobis (2-methylpropionamidine) dihydrochloride]. Photohemolysis was induced using the petri dish monolayer technique, where a volume of 5 mL of each erythrocyte suspension (1%) was placed in the UV-A (315–395 nm) and UV-B (280–315 nm) chambers under controlled temperature (18 ± 1 °C) and different exposure times (60 and 120 min) [32,33]. After irradiation, the RBC suspension was centrifuged at 2000× *g* for 10 min. Two milliliters of supernatant was taken and mixed with 2 mL of Drabkin’s solution (KCN, 0.05 g; K3Fe(CN)6 0.2 g in distilled water). After 10 min of rest, 300 µL was measured at 540 nm in a microplate reader (Hetherington and Johnson, 1984). In addition to this, blood susceptibility was also analyzed via AAPH-induced hemolysis. This molecule was used as a free radical initiator to induce hemolysis on human erythrocytes. To carry out this part, we mixed 100 μL of RBC (2%) + 100 μL of PBS (0.15 M; pH 7.4) + 100 μL of AAPH (40 mM at pH 7.4). After the incubation period of three hours, 1 mL of PBS was added to each sample and centrifuged for 10 min at 2000× *g*. One milliliter of supernatant was taken and mixed with 1 mL of Drabkin’s solution. The reaction was allowed to stand for 10 min under dark room conditions. Later, 300 µL was measured at 540 nm in a microplate reader (Hetherington and Johnson, 1984). Blood susceptibility results are reported as released hemoglobin concentration (g/dL) calculated via the following Equation (3):

(3) $$Released\;hemoglobincon\;centration\;[Hb](\frac{g}{dL})=\frac{A_{Sample}-A_{Blank}}{A_{Pattern}-A_{Blank}}\times 10$$

#### 2.5.4. Antihemolytic Activity Assay

The AAPH-induced hemolysis inhibition was determined via an antihemolytic activity assay [34] on different blood group types. The AAPH molecule was used as a free radical initiator to induce hemolysis on human erythrocytes. Three different reactions were prepared: as a sample reaction, the amounts of 100 μL of RBC (2%) + 100 μL of antioxidant + 100 μL of AAPH (40 mM at pH 7.4) were mixed; as a negative control, the amounts of 100 μL of RBC (2%) + 200 μL of PBS (0.15 M; pH 7.4) were mixed; as a positive control, the amounts of 100 μL of RBC (2%) + 100 μL of PBS (0.15 M; pH 7.4) + AAPH (40 mM at pH 7.4) were mixed. All reactions were incubated for 3 h at 37 °C. After the incubation, 1 mL of PBS was added to each sample and centrifuged for 10 min at 2000× *g*. A volume of 300 µL of supernatant was read at 540 nm on a 96-well microplate (Multiskan Go, Thermo Scientific, Waltham, MA, USA). The results were reported as hemolysis inhibition (%) calculated via the following Equation (4):

(4) $$\%\;HI=\left(\frac{AAPH1-HS}{AAPH1}\right)\times 10$$

where AAPH1 = optical density of the hemolysis caused per radical AAPH, *% HI* = percentage of hemolysis inhibition and HS = optical density of the hemolysis inhibition by each treatment.

#### 2.5.5. In Vitro Photostability Studies

The photoprotective effects of FXN, β-Car, GA, QUE and AA against photo-oxidation induced by UV-A and UV-B radiation were evaluated on human erythrocytes with different blood groups, using the monolayer technique in a petri dish [11,35]. One milliliter of blood (1%) was poured into a petri dish; then, 1 mL of physiological solution and 1 mL of each bioactive compound was added (in different petri dishes). Samples were subjected to preincubation at 37 °C for 30 min. Subsequently, the petri dishes with the mixtures were placed inside the UVR chambers to evaluate the photoprotective effect. As a negative control, 1 mL of erythrocyte suspension (1%) + 2 mL of PBS without exposure to UVR was used. As a positive control, 1 mL of erythrocyte suspension (1%) + 2 mL of PBS (positive control) exposed to UVR was used. The positive control and the samples were exposed to different times (0, 30, 60, 120 and 150 min) of irradiation. After UVR exposure, controls and samples were centrifuged (2000× *g* at 4 °C for 10 min). Subsequently, 300 µL of supernatant was read at 540 nm in a 96-well microplate reader. Results were reported as photohemolysis inhibition (%) calculated via the following Equation (5):

(5) $$\%\;of\;Photohemolysis\;inhibition=\frac{A_{+ve\;control}-{(A}_{Sample}-A_{-ve\;control})}{A_{+ve\;control}}\times 100$$

where A_+ve control_ = absorbance UVR-induced hemolysis, A_−ve control_ = absorbance without hemolysis and A_sample_ = optical density of the photohemolysis inhibition by each treatment.

Finally, to observe RBC’s cellular membrane changes induced by UVR, optical microscopy (100× Eclipse FN1 microscope) was used. For this study, the supernatant samples were performed immediately after reading. Approximately 50 μL of fresh plasma was added to the globular package (from inhibition hemolysis test) and carefully mixed to avoid mechanical damage to the membranes. A blood suspension drop was spread over the slide, creating a thin layer of RBC. To visualize the structure of erythrocyte membranes, it was necessary to stain the RBC via the Wright method [12]. The micrographs were observed with a 100× magnification and presented with a scale bar of 5 µm to compare the RBC size, and the images were captured using the software NIS-Elements F v4.11.0 (Nikon Instruments Inc, Americas, New York, NY, USA; https://www.microscope.healthcare.nikon.com/es_AMS/products/software/nis-elements/viewer, accessed on 30 November 2023).

### 2.6. Statistical Analysis

For this study, all data were analyzed using the statistical program JMP software v16 for Mac and expressed as mean ± SD (standard deviation). One- and two-way ANOVA was performed to observe the interaction between the different factors evaluated. The Tukey test was applied at a *p* < 0.05 confidence interval. The study was carried out under controlled conditions with a minimum of three repetitions (n ≥ 3) for each analysis.

## 3. Results and Discussion

### 3.1. Antioxidant Capacity Assay

In order to determine the antioxidant properties of FXN, β-Car, GA, QUE and AA, three in vitro assays were employed, specifically, FRAP, DPPH• and ABTS•^+^, which can be seen in Table 1. Gallic acid showed a greater reducing power (5970.45 ± 230.07 µmol TE/g) than the other antioxidants tested, transferring electrons to reduce ferric ions to ferrous ions (Fe ^3+^ → Fe ^2+^). Ascorbic acid is an antioxidant capable of preventing cellular oxidation by inhibiting free radicals (3). This characteristic is reflected in the results, promoting the reduction of DPPH• and ABTS•^+^ free radicals, showing an antioxidant capacity of 4190.9 ± 155.16 and 3412.22 ± 21.94 µmol TE/g. Quercetin showed a similar antioxidant potential (3980.61 ± 222.58 µmol TE/g) to ascorbic acid for inhibiting uncontrolled oxidative discharges released by DPPH. Carotenoids such as β-Carotene and fucoxanthin are considered powerful antioxidants. However, previous studies consider that the chemical structure and inhibition mechanism of carotenoids limit their ability to inhibit the DPPH• radical [36].

In this study, FRAP, ABTS•^+^ and DPPH• assays were used to corroborate the antioxidant properties of FXN, xanthophyll; β-Car, carotene; GA, phenol; QUE, flavonoid and AA, ascorbic acid, with the purpose of relating the results with erythroprotective potential. These antioxidants have a system of conjugated double bonds and/or hydroxyl functional groups (-OH) with the ability for electron and hydrogen transfer to reactive molecules and to reduce molecules in an oxidized state to a reduced state [37]. Electron transfer is carried out in place during the second step of Single Electron Transfer, followed by proton transfer (SET-PT) and Sequential Proton Loss Electron Transfer (SPLET) mechanisms. Both mechanisms are carried out in two steps, which are processes in which an electron (e-) is lost and a less reactive cation-radical is formed, while in the second step, the cation-radical is deprotonated to form the corresponding radical [5,6].

It is important to mention that the use of different methodologies to measure antioxidant capacity is a very useful tool. However, ABTS•+ and DPPH• are radicals used within an antioxidant–radical system, while the AAPH molecule is a generator of free radicals, which, being thermolabile, break down into alkoxyl and peroxyl radicals used to evaluate antioxidant capacity in human blood cells. These radicals, generated directly, impact cell membranes, inducing lipid peroxidation and proteolysis [16]. Therefore, the results obtained in this section are very important for explaining erythroprotective potential. In later studies, the antioxidants tested could form an active part of a drug or a nutraceutical or be added to a functional food. For this reason, it is necessary to study its erythroprotective potential (blood biocompatibility, membrane stabilization, photoprotector and antihemolytic effect).

### 3.2. Erythroprotective Potential

#### 3.2.1. Blood Biocompatibility

**Figure 1:**
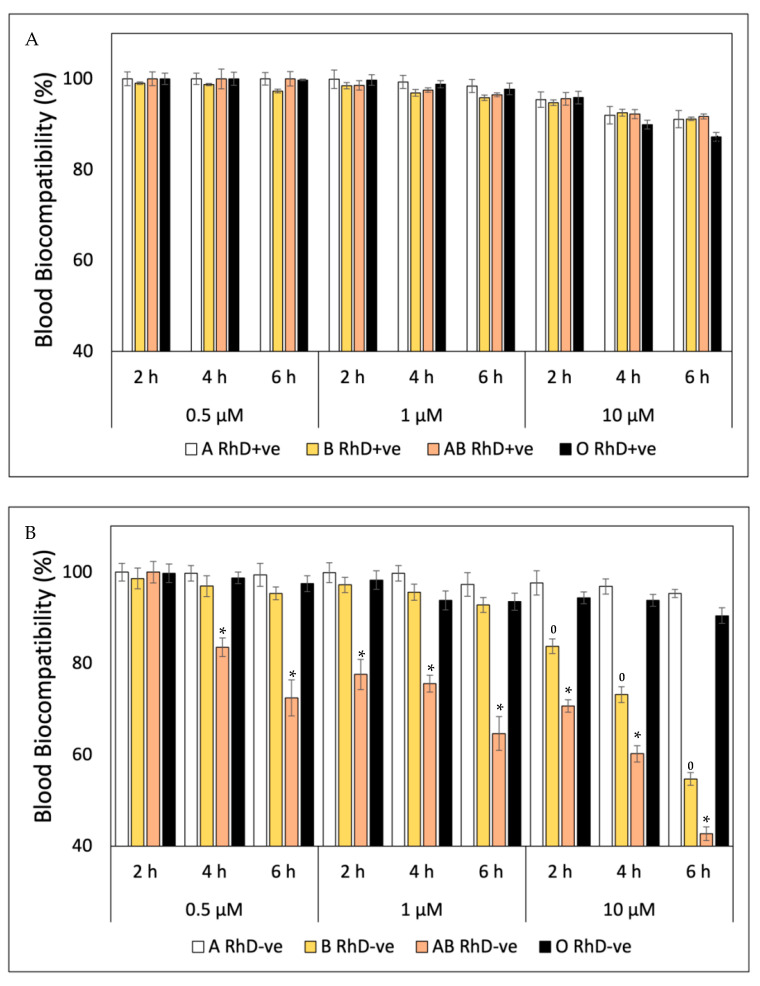
Blood biocompatibility (%) of fucoxanthin on different blood groups ABO RhD+ve (**A**) and RhD-ve (**B**) at different concentrations (0.5, 1.0 and 10 µM) and exposure times (2, 4 and 6 h). All data were analyzed via two-way ANOVA with the interaction of three factors (concentration of fucoxanthin; exposure times; blood groups). * *p* < 0.001, blood group most affected compared to other blood phenotypes by concentration. **^0^** *p* < 0.001, in comparison to the most affected blood group by concentration. Barr represents standard deviation of at least three repetitions (n > 3).

FXN, β-Car, GA, QUE and AA are very important bioactive compounds that promote essential health benefits. All compounds exhibit important antioxidant properties that inhibit cellular oxidation and premature aging induced by exposure to free radicals [14,38,39,40,41]. The blood biocompatibility of FXN decreases at higher concentrations (10 µM) and prolonged exposure times (6 h), showing values around 97 to 100% on red blood cells (RBC) with antigen D (RhD+ve) on the erythrocyte surface (Figure 1A). The O RhD+ve group is most susceptible to the presence of FXN (10 µM, 6 h), with values of 87.20%. At the same times, this trend is observed in the RBC without antigen D (Figure 1B), where the fucoxanthin is more biocompatible with blood groups A and O RhD-ve (10 µM, 6 h), with values above 90%. However, the fucoxanthin significantly (*p* < 0.05) affects blood groups AB and B RhD-ve in the same conditions (10 µM, 6 h). The decrease in the blood biocompatibility of FXN in the AB RhD-ve group is progressive, with values from 83.50% (4 h) to 42.70% (6 h) at 0.05 µM to 10 µM, respectively. However, blood group B RhD-ve presents values from 83.75 (2 h) to 54.19 (6 h) only at 10 µM.

**Figure 2:**
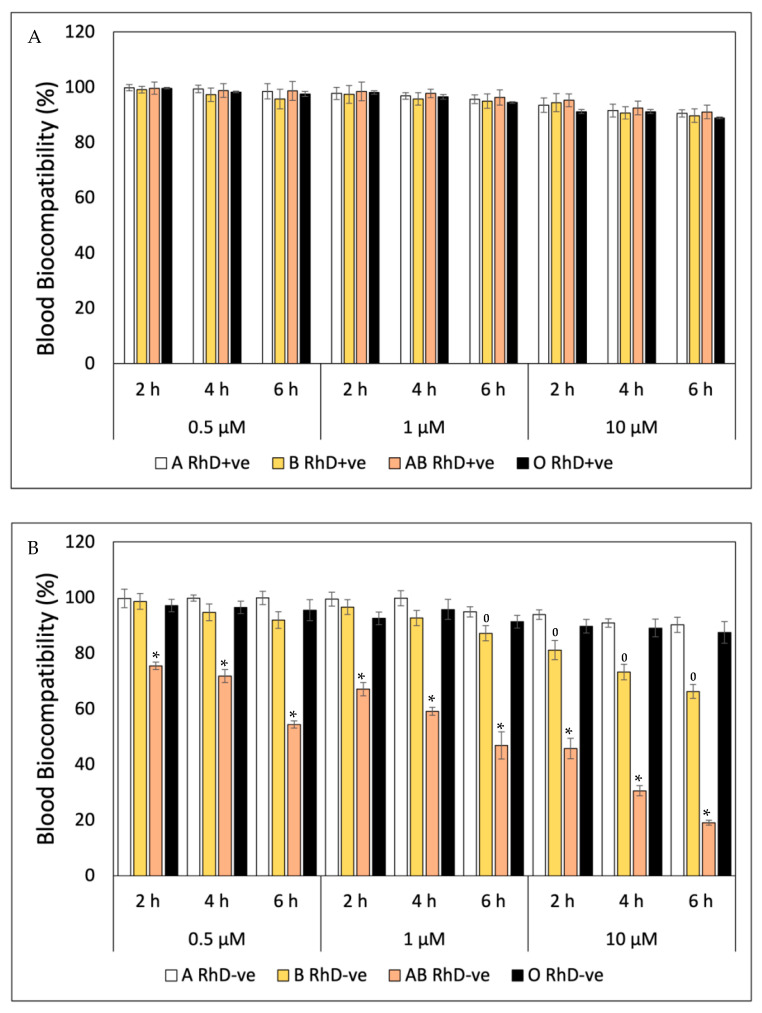
Blood biocompatibility (%) of β-Carotene on different blood groups ABO RhD+ve (**A**) and RhD-ve (**B**) at different concentrations (0.5, 1.0 and 10 µM) and exposure times (2, 4 and 6 h). All data were analyzed via two-way ANOVA with the interaction of three factors (concentration of fucoxanthin; exposure times; blood groups). * *p* < 0.001, blood group most affected compared to other blood phenotypes by concentration. **^0^** *p* < 0.001, in comparison to the most affected blood group by concentration. Barr represents standard deviation of at least three repetitions (n > 3).

As can be seen in Figure 2A, β-Car presented high blood biocompatibility on ABO RhD+ve blood groups, being practically harmless, keeping erythrocyte membranes stable at the concentrations and exposure times tested. However, β-Car presented a cytotoxic effect for certain RhD-ve blood groups (Figure 2B), specifically in groups B and AB RhD-ve. From the first hours, β-Car decreased in biocompatibility with the AB RhD-ve group at different concentrations, with values that ranged from 74.48 to 19.06%. β-Car was cytotoxic for group B RhD-ve at 1 µM at the sixth hour; from this part of the experiment, its biocompatibility decreased after increasing the concentration to 10 µM.

**Figure 3:**
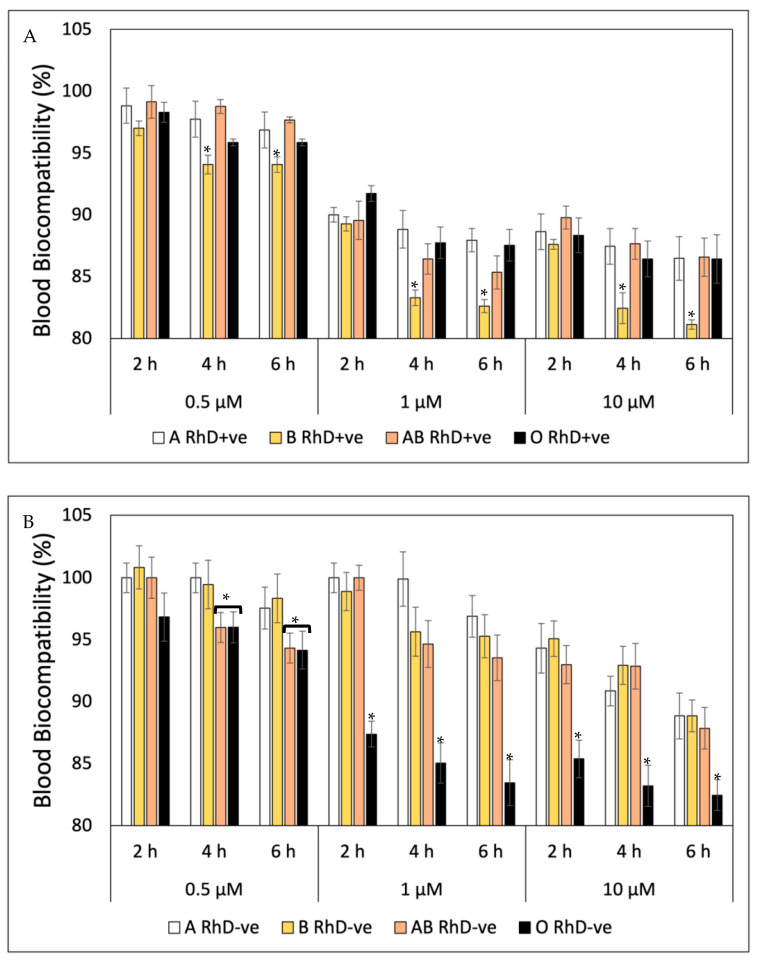
Blood biocompatibility (%) of gallic acid on different blood groups ABO RhD+ve (**A**) and RhD-ve (**B**) at different concentrations (0.5, 1.0 and 10 µM) and exposure times (2, 4 and 6 h). All data were analyzed via two-way ANOVA with the interaction of three factors (concentration of fucoxanthin; exposure times; blood groups). * *p* < 0.001, blood group most affected compared to other blood phenotypes by concentration. Barr represents standard deviation of at least three repetitions (n > 3).

Unlike the blood biocompatibility shown by FXN and β-Car, GA fluctuated from the start of the study (Figure 3). The blood groups non-B RhD+ve presented the highest biocompatibility during the six hours of exposition at three concentrations tested (Figure 3A). At the same time as the concentration of gallic acid increased, the percentage of blood biocompatibility decreased, significantly affecting group B RhD+ve from 4 h at concentrations of 1 µM and 10 µM (83 and 81%, respectively). However, the blood biocompatibility of GA in the RhD-ve groups (Figure 3B) was different. Here, the GA showed higher biocompatibility values in non-O RhD-ve groups. Unlike the groups with the D antigen present in the membrane, the O RhD-ve group was more susceptible to the presence of gallic acid (82.46%). Derived from this, the B RhD+ve and O RhD-ve groups were the most susceptible to hemolysis due to the presence of gallic acid. Probably, the presence or absence of the D antigen is important for the blood biocompatibility of bioactive compounds. However, the cytotoxic mechanism of antioxidants related to erythrocyte surface antigens has not been elucidated. More studies will be necessary on the degree of cytotoxicity in the presence of the D antigen via flow cytometry and FACS analysis using Annexin V-FITC to analyze the erythrocitary apoptotic mechanisms (eryptosis) induced by the cytotoxic action of antioxidant compounds [40]. This toxicity is probably related to the pro-oxidant action of certain antioxidants under very specific conditions and dependent on components of the cell membranes and intracellular signaling that could be involved in the toxicity process. Genetic studies are additional studies related to the expression of genes involved in the synthesis of erythrocyte surface antigens. To accomplish this, a multiparametric analysis would allow us to identify cells in the eryptosis phase through the action of apoptotic genes. These methods could indicate whether the damage to erythrocytes is irreversible, i.e., evaluate their viability after the pro-oxidant action of antioxidants and even exposure to free radicals [39].

**Figure 4:**
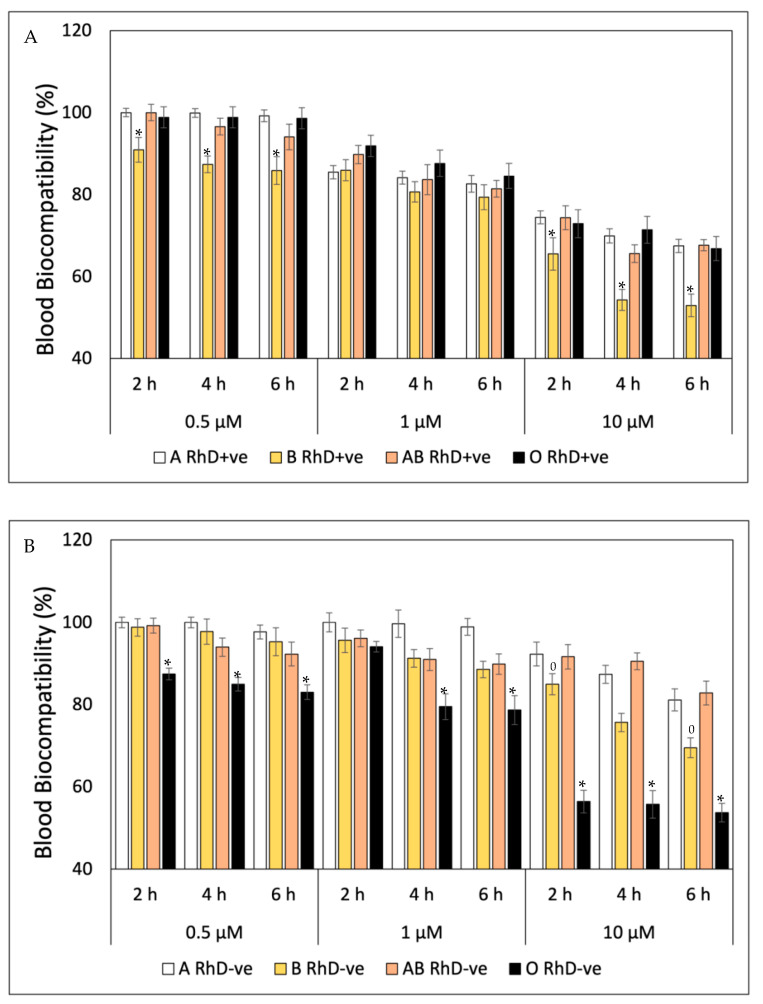
Blood biocompatibility (%) of quercetin on different blood groups ABO RhD+ve (**A**) and RhD-ve (**B**) at different concentrations (0.5, 1.0 and 10 µM) and exposure times (2, 4 and 6 h). All data were analyzed via two-way ANOVA with the interaction of three factors (concentration of fucoxanthin; exposure times; blood groups). * *p* < 0.001, blood group most affected compared to other blood phenotypes by concentration. **^0^** *p* < 0.001, in comparison to the most affected blood group by concentration. Barr represents standard deviation of at least three repetitions (n > 3).

**Figure 5:**
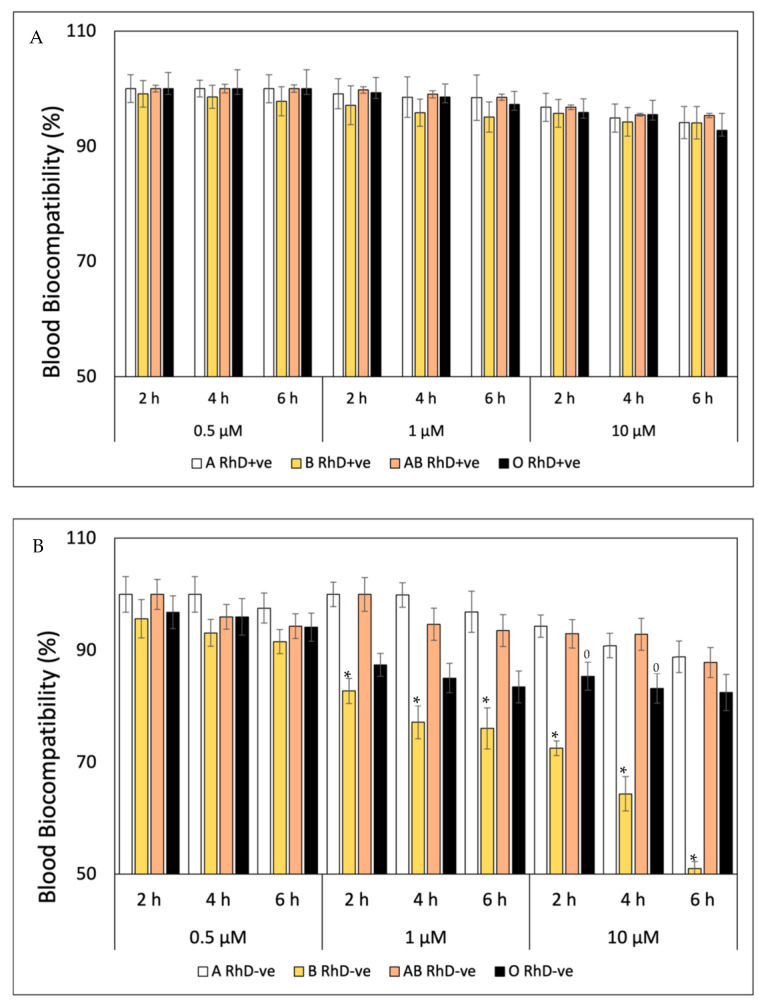
Blood biocompatibility (%) of ascorbic acid on different blood groups ABO RhD+ve (**A**) and RhD-ve (**B**) at different concentrations (0.5, 1.0 and 10 µM) and exposure times (2, 4 and 6 h). All data were analyzed via two-way ANOVA with the interaction of three factors (concentration of fucoxanthin; exposure times; blood groups). * *p* < 0.00, blood group most affected compared to other blood phenotypes by concentration. Barr represents standard deviation of at least three repetitions (n > 3). **^0^** *p* < 0.001, in comparison to the most affected blood group by concentration. Barr represents standard deviation of at least three repetitions (n > 3).

In the case of the blood biocompatibility of quercetin, the results are shown in Figure 4. The blood biocompatibility of quercetin depends on the concentration and the exposure time. Throughout the study, the non-B RhD+ve groups presented the highest values for each concentration (94–100%; 81–91% and 66–71% for 0.1 µM, 1 µM and 10 µM, respectively). Therefore, the group most susceptible to cell damage by quercetin is B RhD+ve, with the greatest decrease in its biocompatibility after the fourth hour at 10 µM. As can be seen in Figure 4B, non-O RhD-ve blood groups presented values of 92 to 100% of blood biocompatibility at concentrations of 0.5 µM. Meanwhile, the O RhD-ve group was the most susceptible to oxidation due to the pro-oxidant actions of quercetin, decreasing its biocompatibility up to 53%. Ascorbic acid is considered innocuous and biocompatible in the four phenotypes of the RhD+ve blood groups at the concentrations and exposure times tested. The values oscillated between 92 and100% biocompatibility (Figure 5A). In contrast, the effects on blood group B RhD-ve were different (Figure 5B), where the values decreased up to 50%, since this blood type is more susceptible to concentrations of 1 and 10 µM of AA. However, the non-B RhD-ve groups maintained around 82 to 94% biocompatibility under the same conditions.

Various studies report that the use of FXN, β-Car, GA, QUE and AA for human consumption could be safe, since it does not present toxicity or mutagenicity at low doses in animal models [27,35,41,42,43,44,45,46]. It has been reported that the levels of these antioxidants in human plasma range may not be sufficient in blood plasma to fully exert the biological power of this molecule [17,28]. FXN presented acceptable blood biocompatibility (>95%) at the tested concentrations of 0.5, 1 and 10 µM. These results are favorable, because it has been reported that the carotenoids levels in human plasma range from 0.1 to 0.9 µM [39]. Like fucoxanthin, β-Car is a very important antioxidant pigment within the carotenoid family. Toxicological studies of β-Car do not exhibit mutagenicity or embryotoxicity through the Ames test and mouse bone marrow cell micronucleus test or embryotoxicity in rats and rabbits [17,44]. However, the impact of the blood biocompatibility of β-Car on different blood types has not been reported. Therefore, our study is one of the first to document the blood biocompatibility of β-Car on human erythrocytes with different surface antigens of the ABO and RhD system.

On the other hand, the concentration considered safe for the consumption of gallic acid is 0.6 µM. Therefore, its toxicity on groups B RhD+ve and O RhD-ve could be due to its pro-oxidant effects at high concentrations. Our study reveals the probability that the administration of high doses of GA could cause adverse health effects. In contrast, the available literature states that the dietary intake of QUE does not produce adverse health effects. On the contrary, when quercetin is consumed in the diet, it prevents premature aging. Polyphenols such as quercetin are a group of phytochemicals that consist mainly of phenolic rings. Its structural characteristics define it as a flavonoid with antioxidant properties that provide cellular protection against oxidative damage. This characteristic reduces cell death induced by free radicals and confers antiproliferative, anticancer, antidiabetic and antiviral effects. It is even known that quercetin plays a role as a healing agent in neurodegenerative pathologies since it easily crosses the blood–brain barrier due to its lipophilic properties [47,48]. On the other hand, it is known that quercetin has a protective effect on human erythrocytes in a model of aging induced by D-Galactose (D-Gal), effectively preventing oxidative damage to membrane lipids through the B3 protein [49].

Like quercetin, ascorbic acid has no cytotoxic effect at concentrations of 60 µM [27]. Ascorbic acid can protect against oxidative damage caused by oxidative stress in cells. The results obtained in our study indicate that blood biocompatibility decreases depending on the bioactive compound evaluated, exposure time and finally the blood group studied. The predominant blood biocompatibility occurred mainly in group A regardless of the RhD, where the most affected groups were blood groups B RhD+ve and O RhD-ve. The increase in antioxidant concentrations promotes an increase in their pro-oxidant potential, acting as oxidizing agents and inducers of cell death.

#### 3.2.2. Membrane Stabilization Assay

**Figure 6:**
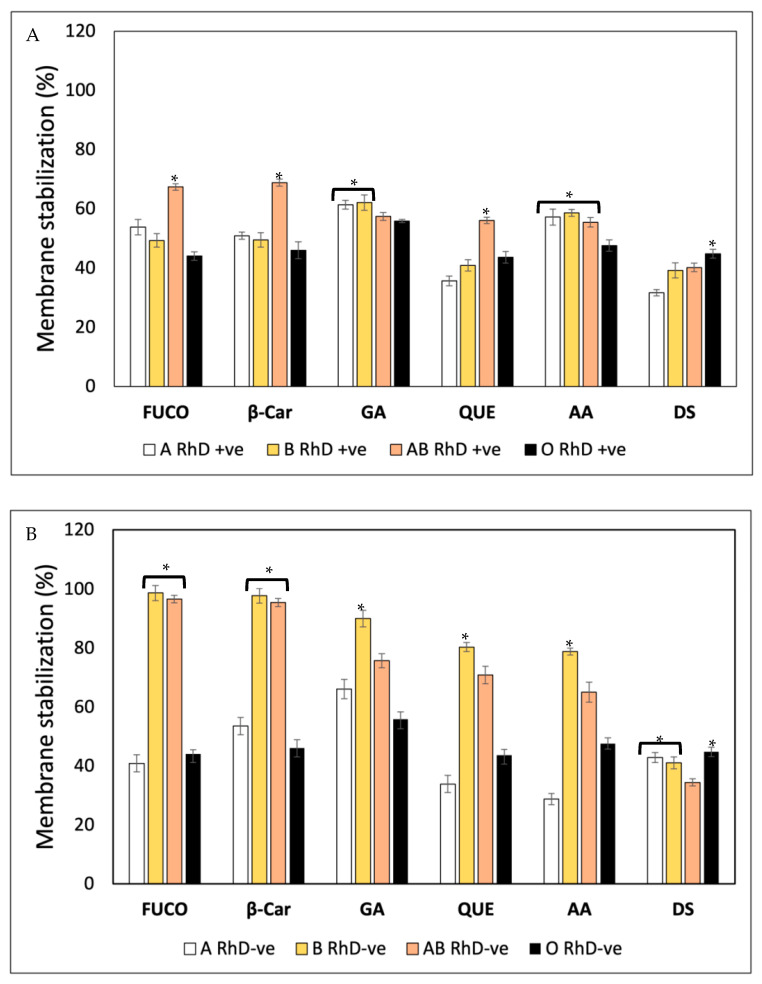
Effect of heat-induced hemolysis for evaluating membrane stabilization (%) from FXN, β-Car, GA, QUE and AA on different ABO RhD+ve (**A**) and RhD-ve (**B**) blood groups at a concentration of 10 µM. All data were analyzed via two-way ANOVA with the interaction of two factors (antioxidant compound; blood groups). * *p* < 0.001, blood group most affected compared to other blood phenotypes by concentration. Barr represents standard deviation of at least three repetitions (n > 3).

The membrane stabilization assay was used as part of the evaluation of the erythroprotective potential of fucoxanthin on ABO and RhD blood groups and evaluated via heat-induced hemolysis and a hypotonicity-induced hemolysis assay. The erythrocytes were exposed to different hemolysis conditions and thermal and osmotic disruptions to determine the percentage of membrane stabilization conferred by FXN, β-Car, GA, QUE and AA and its impact on the different blood groups (Figure 6).

A heat-induced hemolysis assay was carried out to evaluate the membrane stabilization (%) of FXN, β-Car, GA, QUE, AA and diclofenac sodium (DS) as a standard control for membrane stabilization (Figure 6A). Fucoxanthin, β-Car and QUE were more effective in stabilizing the membrane of AB RhD+ve erythrocytes (67.34 ± 2.65, 68.82 ± 2.43 and 56.05 ± 1.07%, respectively), inducing an erythroprotective effect against heat-induced oxidative damage. Gallic acid provided a more protective effect on groups A and B RhD+ve (61–62%) than on AB and O RhD+ve (57–55%). Meanwhile, ascorbic acid stabilized the most non-O RhD+ve blood groups (55–57%). Despite the fact that diclofenac sodium is a potent inflammatory agent capable of stabilizing the lysosomal membrane, the erythroprotective effect was the lowest among all the compounds tested, resulting in the O RhD+ve group (44%) having higher membrane stabilization values than the non-O RhD+ve blood groups (31–40%). Figure 6B shows the membrane stabilization (%) of ABO RhD-ve blood groups. The increase in membrane stabilization in ABO RhD-ve blood types is evident. The antioxidant compounds FXN, β-Car, GA, QUE and AA had a significantly higher protective effect against heat-induced oxidative damage in blood groups B and AB RhD-ve, with values of 55–98%. The erythroprotective effect of diclofenac sodium was diminished compared to antioxidants. The values varied between 42 and 44% in the non-AB RhD-ve groups.

**Figure 7:**
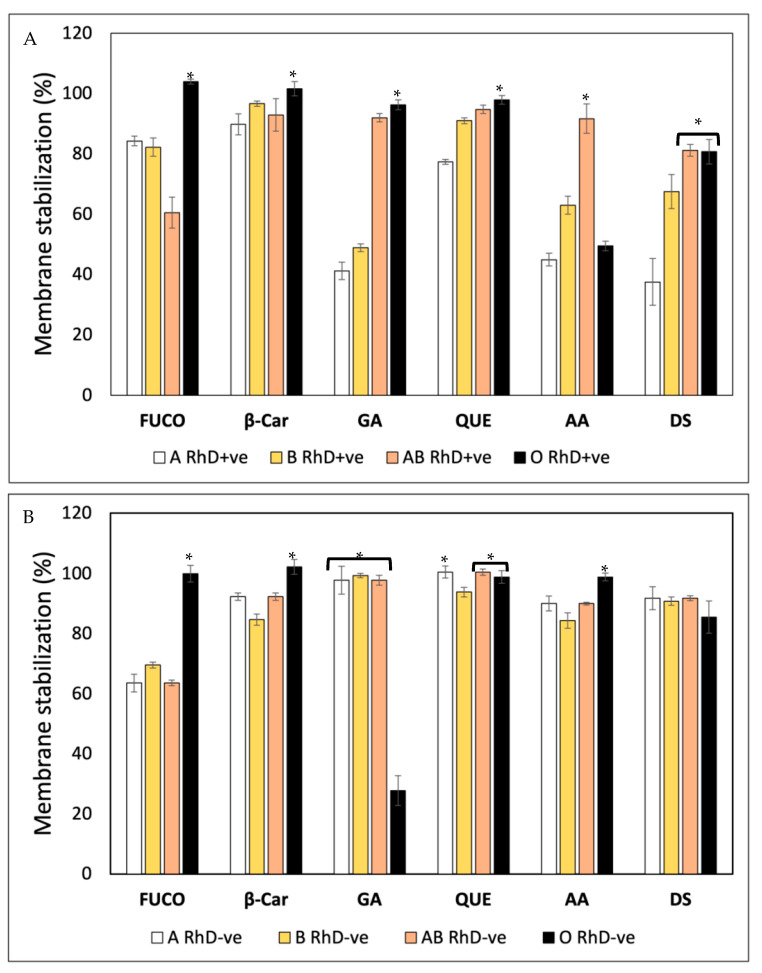
Effect of hypotonicity-induced hemolysis for evaluating membrane stabilization (%) from FXN, β-Car, GA, QUE and AA on different ABO RhD+ve (**A**) and RhD-ve (**B**) blood groups at a concentration of 10 µM. All data were analyzed via two-way ANOVA with the interaction of two factors (antioxidant compound; blood groups). * *p* < 0.001, blood group most affected compared to other blood phenotypes by concentration. Barr represents standard deviation of at least three repetitions (n > 3).

A hypotonicity-induced hemolysis assay was carried out to evaluate the membrane stabilization (%) properties of FXN, β-Car, GA, QUE, AA and DS on ABO blood groups (Figure 7). Figure 7A shows that membrane stabilization (%) depends on both the blood groups and the bioactive compounds. FXN, β-Car, GA and QUE stabilized the erythrocyte membrane efficiently in O RhD+ve groups (97–100%). The biological activity of FXN decreased in the AB RhD+ve group (61%). A notable decrease was observed in group A RhD+ve with GA (41%). The erythroprotective effect of AA increased in the AB RhD+ve group (91%). Finally, the DS drug was effective in the AB and O RhD+ve groups, with approximate values of 81%. The ability of FXN and β-Car to stabilize the membrane was found to be significantly higher in the O RhD-ve group (100%), while in the non-O RhD-ve groups, values of 63–69% were observed for FXN and 84–92% for β-Car (Figure 7B). In contrast, gallic acid did not provide potent stabilizing activity, resulting in a significantly lower value of α in the O RhD-ve group (27.78%). However, its protective effect stabilizing the membranes in non-O RhD-ve groups was high, with values around 93 and 100%. The percentage of membrane stabilization was similar for QUE, AA and DS in all RhD-ve blood groups. The minimum values presented by these compounds were 84% and the maximum 98%. Therefore, FXN, β-Car and GA were the bioactive compounds that varied the most in their protective activity according to the blood group evaluated. In general, according to the results obtained, the evaluated compounds presented a higher percentage of membrane stabilization in the O RhD+ve and O RhD-ve groups, with the exception of gallic acid.

Red blood cell membranes serve as membrane models, where their ABO and RhD antigens play a very important role. Epidemiological research dictates that blood groups are related to the occurrence of various pathologies. Therefore, using membranes with different antigens within the ABO and RhD system is extremely important for finding drugs and/or nutraceuticals capable of preventing diseases associated with blood groups [50,51]. Therefore, plasma membranes have the function of target cells used to measure the inhibition of indicial lipid peroxidation by various factors (heat, hypotonicity and/or ultraviolet radiation). Establishing the interaction between the antioxidant compounds and cell membranes is of great interest [51]. There is not enough information on how carotenoids and ascorbic acid stabilize the erythrocyte plasma membrane. However, in studies by Ruiz-Cruz et al. [11], they evaluated different extracts derived from the marine microalga Navicula incerta on the stabilization of the erythrocyte membrane. They found that the extracts contained FXN and β-Car. These extracts, rich in carotenoids, presented high values of protection to the membrane against various stimuli that generate free radicals. Meanwhile, through absorption and fluorescence spectroscopy, studies have been carried out on the effects of various phenols and flavonoids (e.g., gallic acid, quercetin, chrysin and morin, among others) on goat erythrocyte membranes [27,41]. In these studies, they do not specify the blood group, nor do they clarify whether it was performed with a single group or it was completely roasted.

Membrane exploration with different functional surface antigens serves as a more realistic system of evaluating the action of new drugs or nutraceuticals that could increase therapeutic action and counteract pathologies associated with blood groups such as cancer. In some studies, it has been demonstrated that the binding of flavonoids to erythrocyte membranes significantly inhibits lipid peroxidation, improving their integrity against osmotic disruption. In addition, they express that the antioxidant and antihemolytic activity of bioactive compounds are related to the stabilization of the erythrocyte membrane. This indicates that the flavonoid binding site could be interacting very close to tryptophan residues in transmembrane proteins. This union plays a very important role in stabilizing membranes and inhibiting oxidation, providing important antioxidant potential. Membrane integrity is closely related to the antihemolytic activity of flavonoids [27,41,52,53]. These studies are really interesting, since they demonstrate the importance of the incorporation of antioxidants for the prevention of osmotic disruption and inhibition of lipid peroxidation. However, in our study, human erythrocytes with different blood groups in the ABO and RhD system were used, which gives us a wide range of results. These indicate the impact of blood groups in order to evaluate membrane stability in the face of various factors that produce cell lysis. Therefore, the evaluation of antihemolytic activity is widely justified for its study as part of the erythroprotective potential of FXN, β-Car, GA, QUE and AA.

#### 3.2.3. Blood Susceptibility Test against Oxidative Stress

Since the discovery of the ABO blood group system, interest has remained in its potential involvement in chronic-degenerative diseases such as cancer and infectious diseases such as COVID-19 (SARS-CoV-2) [53]. The difference in the expression of the ABO blood group’s polymorphic antigens can increase or decrease the susceptibility of the host to different pathologies and infections, due to their diverse functions in cell biology. One of the aims of this research is focused on blood susceptibility tests against ultraviolet radiation-induced oxidative stress and AAPH-induced oxidative stress. This information will be useful for clarifying the erythroprotective potential of fucoxanthin. Therefore, erythrocytes were used as a model cell system to delineate the effects of ultraviolet radiation-induced oxidative stress and AAPH-induced oxidative stress on human cells with different surface antigens of the ABO system [6,13,16].

**Figure 8:**
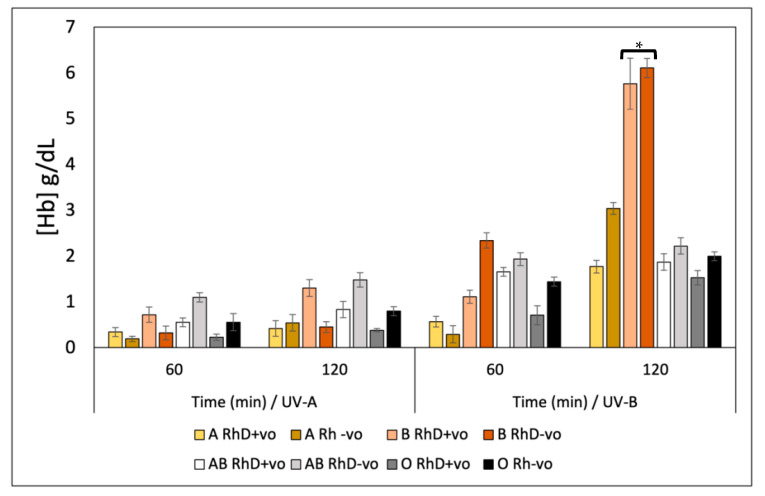
Blood susceptibility study via UV radiation-induced oxidation model. Effect of UV-A (315–395 nm) and UV-B (280–315 nm) irradiation-induced hemoglobin released at different exposure times (30, 60 and 120 min) on ABO and RhD different blood types to evaluate blood susceptibility. All data were analyzed via two-way ANOVA with the interaction of three factors (UVR type; exposure times; blood groups) and their levels. All data were analyzed via two-way ANOVA with the interaction of two factors (antioxidant compound; blood groups). * *p* < 0.001, blood group most affected compared to other blood phenotypes by concentration. Barr represents standard deviation of at least three repetitions (n > 3).

To evaluate the effects of ultraviolet radiation-induced oxidative stress on human erythrocytes, we used the monolayer petri dish technique. It was observed that the oxidative denaturation of the erythrocyte membrane via UVR leads to the release of hemoglobin (Hb) that depends on three factors: type of erythrocyte antigen, type of UV radiation and exposure time (Figure 8). It was established that the groups most susceptible to hemolysis induced by UV-B radiation are the B RhD+ve and B RhD-ve groups, with values of 5.8–6.3 (Hb) g/dL at 120 min of exposure, values significantly higher than those of non-B blood groups, while the B RhD+ve and AB RhD-ve groups were more susceptible to hemolysis against UV-A radiation at 120 min of exposure, with values ranging between 1.2 and 1.4 (Hb) g/dL, where susceptibility is reported as the amount of hemoglobin released by exposure to ultraviolet radiation. At present, the incidence, morbidity and mortality rates of skin cancer are increasing, representing a major public health problem. An exhaustive search has revealed that studies point to ultraviolet radiation as the main etiological agent in the development of skin cancer [15]. Ultraviolet radiation (UVR) causes damage to genetic material, causing DNA mutations, which later lead to the development of neoplasms, in this case, skin cancer. Previous studies have examined the association between ABO blood groups and skin cancer risk induced by prolonged UVR exposure [15].

Specifically, UVR has been shown to be one of the main risk factors for developing non-melanoma skin cancer and squamous cell carcinoma. Studies to date suggest focusing on finding photoprotective compounds for groups A RhD-ve, AB RhD+ve and O (RhD not specified) that inhibit oxidative damage induced by ultraviolet radiation. It has been reported that there is a higher incidence of these types of cancers in populations with the aforementioned blood groups [15]. By detecting in our study that the blood groups B RhD+ve and AB RhD-ve are susceptible to UV-A radiation and B RhD+ve and B RhD-ve are susceptible to UV-B radiation (UV-B being of higher risk), it could be considered that free radicals and the oxidative stress generated by UVR participate in a great way for the development of skin cancer in the risk groups mentioned above, these groups being more susceptible to oxidative damage. Our research is intended to stimulate the prevention of cancer induced by UVR in subjects at higher risk or more susceptible and promote basic research addressing this social problem. The photoprotective effect of the antioxidants studied in our research could help prevent the incidence of skin cancer for the risk groups most affected by UVR. Ultraviolet radiation generates free radicals by exposing tissues to high doses of radiation for a long time.

Generating consequences that affect cellular integrity leads to the appearance of certain chronic pathologies such as skin cancer, tissue injuries and eye damage. However, ultraviolet radiation is not the only source of free radical formation; there are different pathways that generate oxidative stress. Food additives, metals in transition or pesticides induce the formation of free radicals that chemically oxidize lipids and proteins found in the cell membranes of tissues. In this step of the research, the AAPH molecule was used to induce lipid peroxidation and proteolysis on the different erythrocyte phenotypes to evaluate blood susceptibility [54].

**Figure 9:**
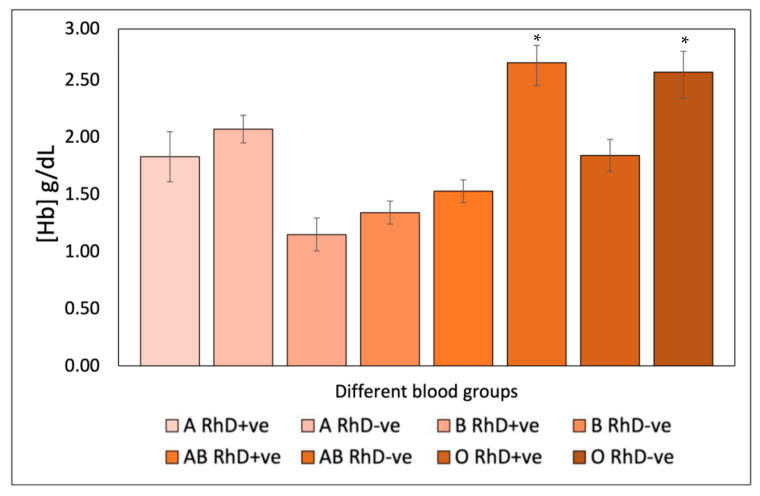
Blood susceptibility study via AAPH-induced oxidation model. Effect of oxidative hemolysis via the free radical generator AAPH as an inducer of hemoglobin release on different ABO and RhD blood types to evaluate blood susceptibility. One-way analysis of Pearson correlation coefficient of the results of oxidative hemolysis (%) tests showed greater variance (ANOVA). Different lowercase letters represent significant differences via one-way ANOVA (*p* < 0.001). * *p* < 0.001, blood group most affected compared to other blood phenotypes by concentration. Barr represents standard deviation of at least three repetitions (n > 3).

Compared with the UV radiation-induced model, the AAPH-induced oxidation model showed lower hemoglobin release (Figure 9). This released hemoglobin is directly proportional to the damage exerted by the uncontrolled oxidative discharges induced by the alkoxyl and peroxyl radicals generated by AAPH. Oxidative stress induced by AAPH affects erythrocyte biochemical processes and induces lipid peroxidation on the erythrocyte membrane; this effect is dependent on ABO and RhD antigens. This chemical oxidation model was not as powerful as the UVR oxidation model. However, it caused irreversible damage to the erythrocytes. Blood groups AB RhD-ve and O RhD-ve were most susceptible to oxidative hemolysis, with values between 2.50 and 2.62 (Hb) g/dL. These results will promote the search for antioxidant compounds with greater potential to inhibit oxidative stress in those groups with phenotypes more susceptible to degradation. In this case, this prevents lipid peroxidation and proteolysis of RhD-ve group B and AB RhD+ve due to chemical agents that induce free radicals such as food additives, heavy metals, pesticides and food preservatives, among others. Thus, this contributes to the prevention of chronic-degenerative pathologies associated with the blood groups of the most susceptible groups [55].

Peroxyl and alkoxyl radicals react with the lipids and proteins of the erythrocyte membrane [16]. The exact mechanisms of interaction between erythrocyte surface antigens and free radicals are unknown. However, it can be observed that the presence of these antigens contributes to reducing or increasing cellular oxidation. The increase in reactive oxygen species leads to the formation of holes in the erythrocyte membrane, causing the release of hemoglobin, especially H_2_O_2_, which, being a product of physiological cellular metabolism during enzymatic and non-enzymatic reactions, triggers the oxidation of membrane components such as lipids and proteins. The chain reactions of ROS promote morphological changes in the cell membrane, compromising the structure and leading to eryptosis. Therefore, cytoprotective antioxidants could be a source of antioxidants capable of reducing oxidative stress associated with blood groups, highlighting their antihemolytic capacity [56,57].

#### 3.2.4. Antihemolytic Activity Assay

Regarding the antihemolytic assay, the AAPH molecule is thermally decomposed into molecular nitrogen and carbon radicals that combine to produce unstable compounds. The products of this reaction are called free radicals; they are mainly composed of peroxyl and alkoxyl radicals. Once the highly reactive molecules come into contact with the erythrocyte plasma membrane, they induce lipid peroxidation and proteolysis. This contact affects the stability of the cell membrane; in the case of the human erythrocyte membrane, it causes changes in erythrocyte morphology, disruption and eryptosis. The action of free radicals generated by AAPH can be stopped by erythroprotective antioxidants. These morphological changes and effects on the erythrocyte membrane can be prevented by using erythroprotective antioxidants, inhibiting AAPH-induced free radicals [58]. Therefore, the aim of this section is to determine the antihemolytic activity of FXN, β-Car, GA, QUE and AA on the different blood groups of the ABO and RhD system.

According to our results, the antihemolytic response of antioxidants directly depends on blood groups with slight differences between antioxidant compounds. The tested antioxidants exerted an erythroprotective effect on RhD+ve blood erythrocytes, above 80% inhibition of hemolysis (Table 2). FXN and β-Car inhibited total hemolysis in the A RhD+ve phenotype (≈100%). Gallic acid, quercetin and ascorbic acid generated a similar erythroprotective potential, around 97–100% inhibition of hemolysis on the AB RhD+ve and AB RhD+ve phenotypes. However, this antihemolytic effect exerted by antioxidants decreased in the O RhD+ve phenotype until it decreased by 80%. This could denote that erythrocytes with the absence of antigens A and B and with the presence of antigen D affect the erythroprotective potential of these antioxidants. On the other hand, it is possible that the presence of antigens A and B (RhD+ve) increases antihemolytic activity. The inhibitory effect of erythroprotective antioxidants decreased in non-O RhD-ve groups, even below 80% in some cases (Table 3). This effect was increased in the RhD-ve group O using fucoxanthin and β-Carotene as erythroprotective agents. The cellular integrity of the B RhD-ve phenotype was preserved above 80% using quercetin and ascorbic acid as erythroprotective agents. In this case, the results are more heterogeneous than for the phenotypes with RhD-ve.

Our studies have shown that erythrocyte surface antigens play an important role in the effectiveness of the erythroprotective potential of antioxidants and oxidative stress- induced membrane alterations. Human cells are constantly exposed to endogenous and exogenous sources of reactive oxygen and nitrogen species (RONS), such as nitric oxide, nitrous oxide, peroxynitrite, hydrogen peroxide, superoxide, alkoxyl and peroxyl. The endogenous antioxidant system of red blood cells neutralizes most of the RONS. However, when the production of RONS increases, it is necessary to resort to exogenous antioxidants. FXN, β-Car, GA, QUE and AA are endogenous antioxidants that inhibit free radicals produced by the thermal degradation of AAPH [56,59].

Previously, the literature has reported that the thermolysis of 2,2′-azobis (2-amidinopropane) hydrochloride (AAPH) is a useful model of oxidative stress to evaluate the oxidative susceptibility and antioxidant capacity of biomolecules on erythrocyte membranes. The chemical stress induced by AAPH shows an oxidative mechanism led by alkoxyl and peroxyl radicals that directly and independently oxidize cell membranes. These unstable molecules oxidize proteins and lipids in membranes, stealing electrons from their structures. Here, membrane biomolecules are chemically destabilized, and a chain reaction begins. These trigger oxidation reactions, which continue on the cell surface until inducing erythrocyte cell death. It is worth noting that this does not happen in the presence of antioxidants, since antioxidants interpose between the cell membrane and reactive oxygen species. The double bonds and functional groups of antioxidants transfer electrons or protons to stabilize free radicals, preventing them from oxidizing membrane constituents. This suggests that AAPH may highlight differences in susceptibility with high sequence homology [55,59].

Compared to the AAPH-induced oxidation model, the UV-induced model suggests that light-sensitive molecules are equally sensitive to AAPH. Therefore, AAPH can be used to evaluate oxidation sensitivity in cells in multiple ways. In some studies, AAPH-induced oxidative stress has been shown to affect embryonic development processes associated with vasculogenesis and angiogenesis, causing irreversible damage to the hearts of bird embryos. This oxidation model can not only be applied to red blood cells but also to more complex biological systems. Therefore, reactive products derived from AAPH thermolysis can be used in animal models to elucidate antioxidant mechanisms and their effects on tissue development to protect various organ systems from oxidative stress. In addition to the oxidative model induced by AAPH and ultraviolet radiation, an oxidation model via hydrogen peroxide (H_2_O_2_) has been reported. It has been observed that this model does not induce the oxidation of certain amino acids such as tryptophan. The oxidative model induced by hydrogen peroxide has been used to study oxidative stress in endothelial cells. It has been shown to induce sufficient stress in this type of cell. However, high doses of hydrogen peroxide and exposure times of eight hours are needed to cause a significant oxidative effect. A study by Morabito et al. [56] demonstrated that 30-min exposure to 300 μM H_2_O_2_ reduces SO_4_- absorption, through damage to cytoplasmic components in human erythrocytes. It should be noted that this study did not evaluate the ability of hydrogen peroxide to function as an inducer of cellular oxidative stress. They confirm that in human erythrocytes, band 3 and intracellular content are crucial to maintaining normal function in cells subjected to oxidative stress. The oxidative stress induced by hydrogen peroxide is neutralized in this case by curcumin, which has a powerful erythroprotective effect associated with the functions of band 3. Regardless of the oxidative stress models used, antioxidants are undoubtedly the best compounds for reducing or eliminating free radicals and their uncontrolled oxidative discharge processes [7,53,60].

The erythroprotective potential of biomolecules such as proteins, phycobiliproteins and carotenoids is associated with the presence of blood groups. A study by Remigante et al. [49] verified the protective effect of quercetin on human erythrocytes in an aging model induced by D-Galactose (D-Gal). They observed that quercetin at a concentration of 10 µM has a protective effect on the function of the B3 protein (B3P). They observed that the anion exchange capacity through the B3 protein (B3P) effectively prevents oxidative damage to membrane lipids. The evaluation of B3P function could be a useful tool for monitoring erythrocyte homeostasis; at the same time, B3P is identified as a potential target of antioxidant treatments to counteract uncontrolled oxidative discharges related to aging. Despite this, it is necessary to delve deeper into the erythroprotective action of QUE to determine the effectiveness of using this flavonoid in the pharmacological industry against oxidative stress [47,49].

In our research, it was observed that the protective effect varies depending on the surface antigens. These antigenic receptors are associated with inflammatory responses without pathological stimulus, that is, systemic inflammation that could lead to the development of a neoplastic process [11,13]. The susceptibility of phenotype A to certain pathologies is recurrent. This characteristic could occur in people suffering from different types of cancer (maternal cancer) and infectious diseases (HIV infection, hepatitis B virus and severe acute respiratory syndrome coronavirus 2 (SARS-CoV-2)) related to this blood group. Recently, signs of association have been detected between the 9q34.2 locus of SARS-CoV-2 (COVID-19) and the ABO blood group locus [14]. A specific analysis showed a greater risk of acquiring this infection in blood group A than in non-A groups, causing a greater risk of respiratory failure. Therefore, the possible participation of ABO blood groups in oxidative stress induced by the AAPH molecule is confirmed.

For this reason, our study has focused on determining the erythroprotective potential of fucoxanthin, β-Carotene, gallic acid, quercetin and ascorbic acid, representing biomolecules that belong to different families of antioxidants (carotenes, xanthophylls, phenolic acids, flavonoids and vitamins), with the aim of preventing or reducing oxidative stress induced by different sources. The results indicate that the different antioxidants tested in our research could be potential candidates for providing protection, mainly to cells with antigen A on their membrane. These antioxidants are chemical substances that help neutralize free radicals, unstable molecules that can damage cells and contribute to the development of chronic-degenerative diseases. It has been shown that a diet rich in antioxidants can have beneficial effects on human health. Antioxidants play a very important role in protecting cells against oxidative stress. However, there is emerging research that suggests that the benefits of antioxidants may vary depending on an individual’s blood group [11,12,13].

#### 3.2.5. In Vitro Photostability Studies

##### In Vitro Photoprotection Efficacy

**Figure 10:**
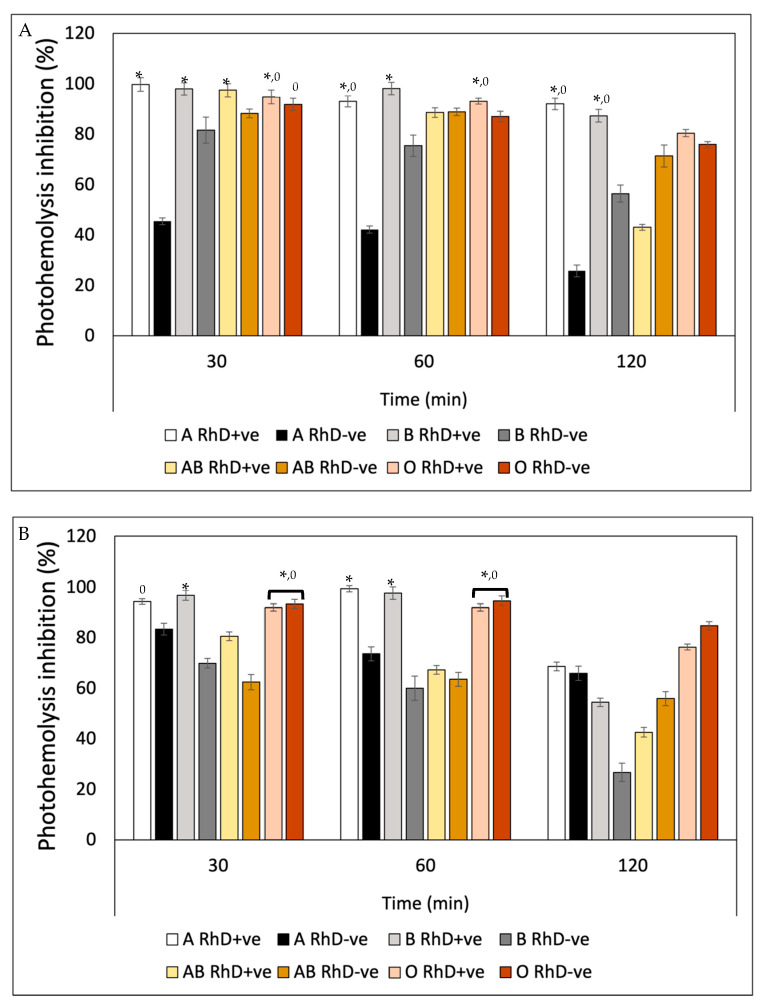
Photoprotector effect of fucoxanthin (FXN) against UV-A (**A**) and UV-B (**B**) irradiation-induced hemolysis on human erythrocytes with different ABO and RhD blood groups at different exposure times (30, 60 and 120 min). All data were analyzed via two-way ANOVA with the interaction of three factors (exposure times; blood groups) and their levels. * *p* < 0.001, blood group most affected compared to other blood phenotypes by concentration. **^0^** *p* < 0.001, in comparison to the most affected blood group by concentration. Barr represents standard deviation of at least three repetitions (n > 3).

Ultraviolet radiation (UVR) induced a substantial decrease in the integrity of the erythrocyte membrane of the eight blood phenotypes, measured through hemoglobin release (Figure 10). After preincubation with fucoxanthin, the levels of photoprotection against UV-A radiation increased significantly in non-A RhD-ve blood groups in the first 60 min of exposure, with values around 75–99% inhibition of photohemolysis (Figure 10A). However, after 120 min, photoprotection decreased in most blood groups (22–80%), except in groups A RhD+ve and B RhD+ve (90–95%). Undoubtedly, the groups most affected by UV-A radiation were groups A RhD-ve and B RhD-ve, presenting a greater susceptibility to cellular oxidation. Particularly interesting, those erythrocytes with the absence of antigen D showed an increase in their photo-oxidation, specifically in antigens A and B, being the phenotypes that provided the least photoprotection from fucoxanthin. Meanwhile, it can be seen from Figure 10B that the photoprotection of fucoxanthin was different against UV-B radiation. Here, photo-oxidation was more evident after 30 min of exposure. Despite this, the inhibition of photohemolysis ranged between 60 and 97% at 30 and 60 min of exposure. In this exposure period, fucoxanthin provided greater photoprotection in blood groups A and B RhD+ve (94–98%). Unlike the results reported for UV-A radiation, UV-B radiation did not drastically affect group A RhD-ve, but rather group AB RhD+ve. At the end of the exposure period (120 min), photoprotection decreased drastically for group B RhD-ve compared to the previous two periods.

**Figure 11:**
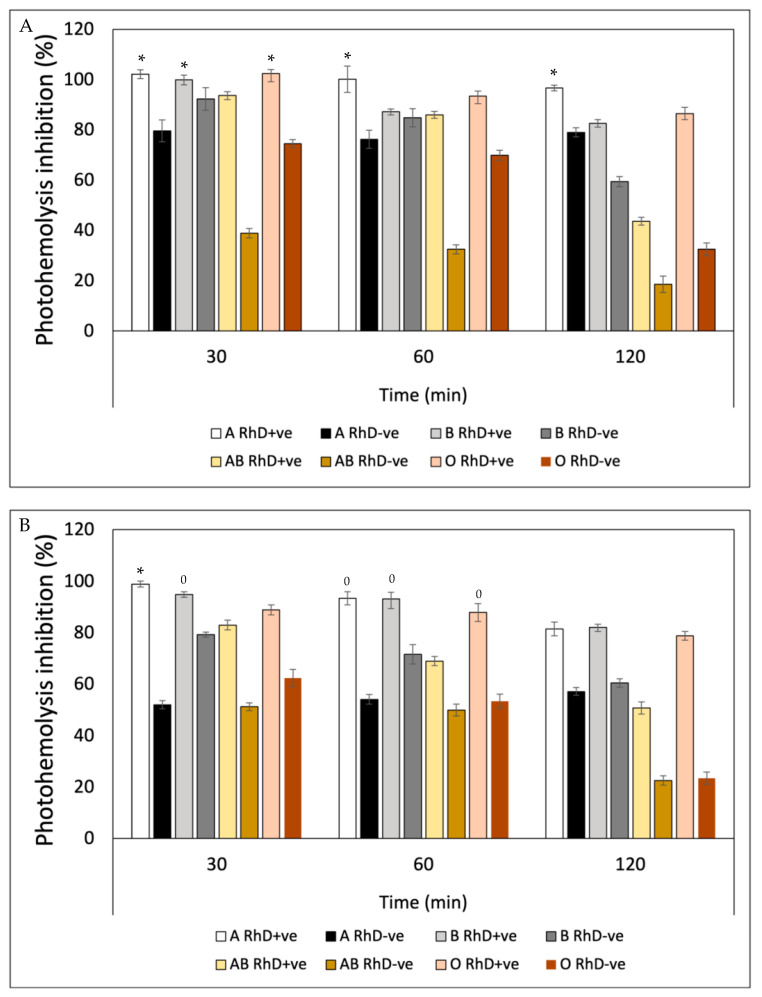
Photoprotector effect of β-Carotene (β-Car) against UV-A (**A**) and UV-B (**B**) irradiation-induced hemolysis on human erythrocytes with different ABO and RhD blood groups at different exposure times (30, 60 and 120 min). All data were analyzed via two-way ANOVA with the interaction of three factors (exposure times; blood groups). * *p* < 0.001, blood group most affected compared to other blood phenotypes by concentration. **^0^** *p* < 0.001, in comparison to the most affected blood group by concentration. Barr represents standard deviation of at least three repetitions (n > 3).

In general, all groups decreased in this period, with a greater photoprotection present in the O RhD-ve group by fucoxanthin (85.34%). The photoprotective effect exhibited by β-Carotene against hemolysis induced by UV-A radiation (Figure 11A) presented similar values in the three exposure periods on the eight erythrocyte phenotypes. β-Carotene conferred a greater photoprotective effect on A RhD+ve erythrocytes compared to the remaining seven phenotypes during the three periods. The values ranged between 95 and 100% inhibition of photohemolysis, while the AB RhD-ve group was the most affected by UV-A radiation despite the photoprotection provided by β-Carotene. This means that erythrocytes with AB antigens with absent RhD probably present an antagonistic effect (17–38%), presenting a significant decrease (*p* > 0.05) with respect to the values presented for groups A and B RhD+ve, A and B RhD-ve and AB RhD+ve. This same trend occurred in blood groups exposed to UV-B radiation (Figure 11B). The most notable difference is found under the 120-min period where the value of the O RhD-ve group decreased to 18.54%.

**Figure 12:**
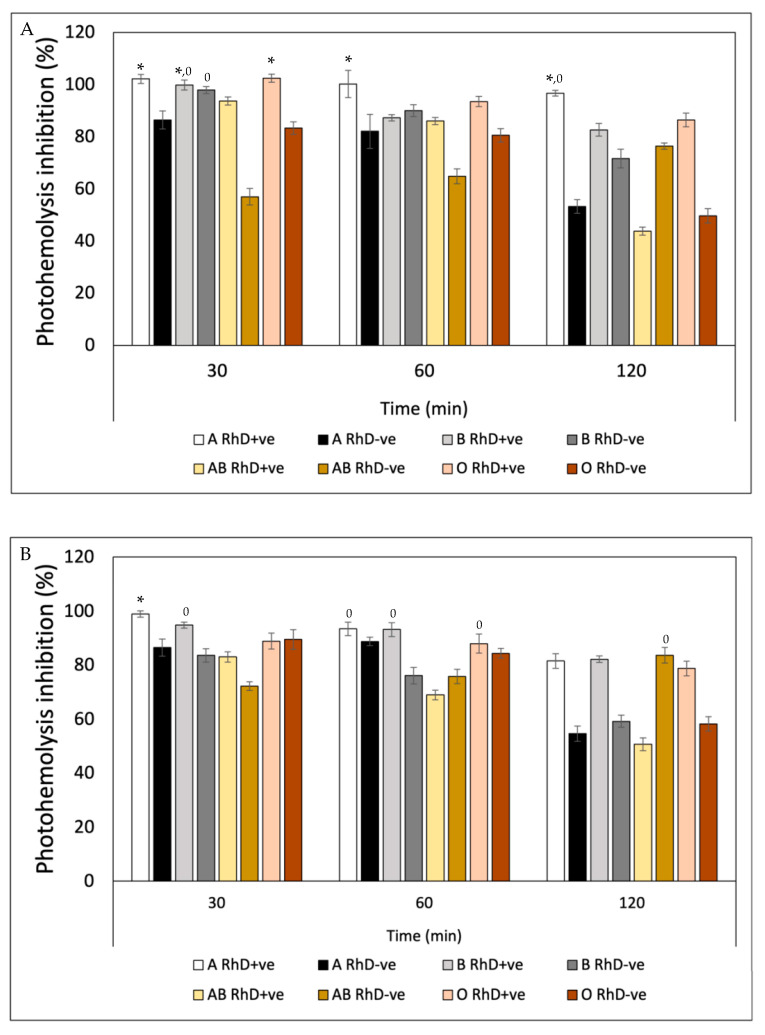
Photoprotector effect of gallic acid (GA) against UV-A (**A**) and UV-B (**B**) irradiation-induced hemolysis on human erythrocytes with different ABO and RhD blood groups at different exposure times (30, 60 and 120 min). All data were analyzed via two-way ANOVA with the interaction of three factors (exposure times; blood groups). * *p* < 0.001, blood group most affected compared to other blood phenotypes by concentration. **^0^** *p* < 0.001, in comparison to the most affected blood group by concentration. Barr represents standard deviation of at least three repetitions (n > 3).

Figure 12 also shows a notable variability in the photoprotective effect of gallic acid on the different erythrocyte phenotypes as well as the previous bioactive compounds. This variability can be observed in the affinity of gallic acid by the different phenotypes seen in Figure 12A. This trend indicates that as the exposure time increases, the photoprotective effect of gallic acid decreases. At the time of 120 min, erythrocyte hemolysis due to exposure to UV-A radiation is accentuated, revealing a decrease in the protective effect in groups A RhD-ve, AB RhD+ve and O RhD-ve, with values ranging between 43 and 51%. Gallic acid showed greater effectiveness in inhibiting photo-oxidation induced by UV-B radiation (Figure 12B) on RhD+ve erythrocytes A and B, with notable photoprotective stability in all exposure periods (80–98%). However, free radicals and oxidative stress induced at 120 min caused cellular degradation, lipid peroxidation and proteolysis on the erythrocyte membrane of the AB and O RhD-ve groups (≈20%). This result indicates that gallic acid probably did not have such a strong affinity with these blood groups, since this decrease in the photoprotective effect was observed from the first 30 min of exposure.

**Figure 13:**
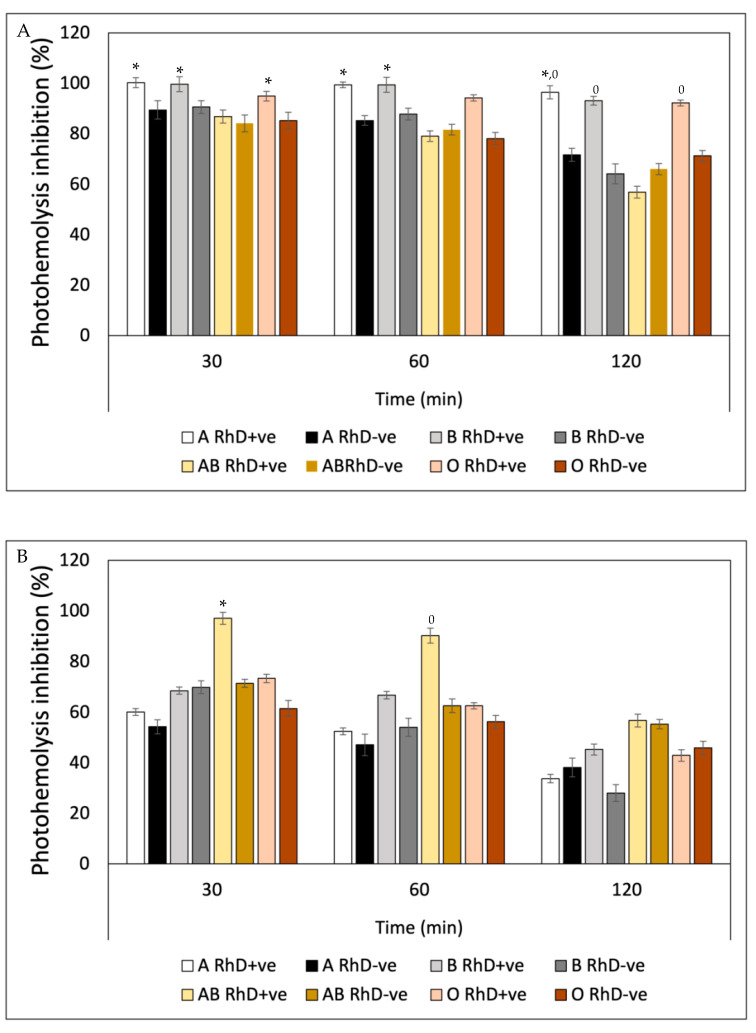
Photoprotector effect of quercetin (QUE) against UV-A (**A**) and UV-B (**B**) irradiation-induced hemolysis on human erythrocytes with different ABO and RhD blood groups at different exposure times (30, 60 and 120 min). All data were analyzed via two-way ANOVA with the interaction of three factors (exposure times; blood groups). * *p* < 0.001, blood group most affected compared to other blood phenotypes by concentration. **^0^** *p* < 0.001, in comparison to the most affected blood group by concentration. Barr represents standard deviation of at least three repetitions (n > 3).

The photoprotective efficacy of quercetin (Figure 13) showed a behavior dependent on blood groups, which means that the inhibitory potential of quercetin on free radicals induced by UV-A and UV-B radiation varied depending on the erythrocyte surface antigen. Figure 13A shows the variation of the effect of quercetin on erythrocyte phenotypes with a very similar behavior for the three exposure periods. A greater response was obtained in groups A, B and O, where antigen D was present in all three group types (90–98%). In Figure 13B, a greater photo-oxidation can be seen with UV-B radiation as opposed to UV-A radiation. Here, the intensity of ultraviolet radiation significantly decreased the photoprotective efficacy of quercetin in the three established time periods. In the first 60 min of exposure, quercetin provided the AB RhD+ve group with an efficacy between 90 and 95%, which decreased after 120 min (58%). Photoprotective efficacy decreased below 60% for all erythrocyte phenotypes.

**Figure 14:**
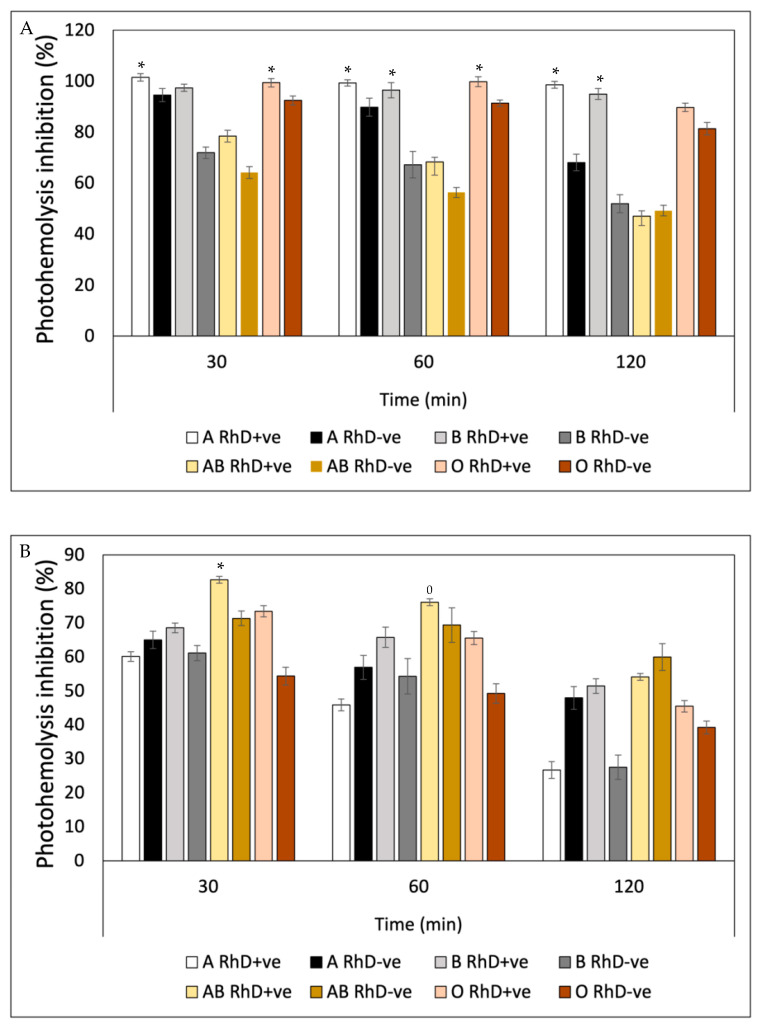
Photoprotector effect of ascorbic acid (AA) against UV-A (**A**) and UV-B (**B**) irradiation-induced hemolysis on human erythrocytes with different ABO and RhD blood groups at different exposure times (30, 60 and 120 min). All data were analyzed via two-way ANOVA with the interaction of three factors (exposure times; blood groups). * *p* < 0.001, blood group most affected compared to other blood phenotypes by concentration. **^0^** *p* < 0.001, in comparison to the most affected blood group by concentration. Barr represents standard deviation of at least three repetitions (n > 3).

The results of the efficacy of ascorbic acid showed that blood groups B RhD-ve, AB RhD+ve and AB RhD-ve were most susceptible to degradation by UV-A radiation (45 and 74%) in the three periods (Figure 14A). The oxidative stress present in these blood groups was evident, when compared to the remaining blood groups. Once again, groups A RhD+ve and B RhD+ve showed a better response to the protection provided by ascorbic acid. Not only this antioxidant but also the previous antioxidants exhibited significant photoprotection against photo-oxidation induced by UV-A radiation on these same blood groups. The evaluation of the sensitivity to UV-B radiation of erythrocytes preincubated with ascorbic acid showed statistically significant differences between each blood group tested (Figure 14B). Ascorbic acid seemed to suppress the effects of UV-B radiation on the erythrocyte membrane of the AB RhD+ve group in the first and second periods (30 and 60 min) of exposure. However, at 120 min, the effects of radiation suppressed the photoprotection provided by ascorbic acid, reducing its effect on all blood groups.

These pathologies occur through the formation of reactive oxygen species (ROS) [61,62]. Fortunately, the skin has endogenous antioxidants that it uses as defense mechanisms to inhibit or delay the oxidative damage generated by UVR. Undoubtedly, this defense system decreases with chronic exposure to ultraviolet rays; therefore, a strategy to remedy this deficiency would be the use of antioxidant phytochemicals capable of delaying or inhibiting cell damage caused by free radicals, providing photoprotection to human skin [63,64]. It has been observed that bioactive compounds such as flavonoids and carotenoids are capable of inhibiting cellular photo-oxidation. However, in the same way as endogenous antioxidants, exogenous antioxidants could be degraded by chronic UVR exposure. A probable hypothesis for reducing the development of pathologies associated with the effects of UVR is the application of oral, skin or ocular antioxidants that eliminate reactive oxidants and modulate the cellular redox state. For this reason, part of our study focused on evaluating the photostability of fucoxanthin, blood susceptibility and in vitro photoprotection efficacy. This study allows for obtaining information on the use of antioxidants as photoprotective agents, inhibiting the effects of UVR on different erythrocyte blood groups as part of the erythroprotective potential [64,65].

Sunlight is the main source of human exposure to ultraviolet radiation, which is classified into three main types: UV-A (315–395 nm), UV-B (280–315 nm) and UV-C (100–280 nm). These subdivisions of electromagnetic energy emit photons that are between the wavelengths of visible light and gamma radiation. Based on its electrophysical properties, each component of ultraviolet radiation can exert harmful effects on cells, tissues and molecules. The predominant UV radiation types in sunlight are UV-A (90–95%) and UV-B (5–10%), while UV-C is absorbed by the atmospheric zone, a characteristic for which this radiation was excluded from our study [66]. UV radiation penetrates the skin depending on the wavelength; the longest wavelength (UV-A) penetrates deeply into the dermis. On the other hand, the long wavelength (UV-B) is almost completely absorbed by the epidermis. This exposure generates free radicals, which are a health risk since they can damage important biomolecules such as DNA through indirect photosensitization reactions. These oxidation reactions cause molecular rearrangements or photoproducts that cause mutations and cancer [67]. There are two pathways of cellular damage mediated by UV radiation: (a) the direct absorption of radiation by cellular components, altering cellular chemistry and the formation of an excited state, and (b) the absorption of radiation by endogenous or exogenous cells that are excited to their triplet states, a mechanism called photosensitization [35,68,69].

Endogenous photosensitization is responsible for inducing reactive oxygen species by UVR, generating superoxide anion and singlet oxygen. Over time, through different mechanisms, it is possible to induce the synthesis of other reactive oxygen species, such as peroxyl radicals, hydrogen peroxide and hydroxyls. In this context, various studies associate the generation of free radicals induced by UVR with skin pathologies. Fortunately, the skin and the human body have an elaborate defense system of endogenous antioxidants capable of mitigating the effects of oxidative stress induced by chemical agents, cellular metabolism, inflammatory processes and external agents such as UVR. However, excessive exposure to ultraviolet radiation can reduce the skin’s antioxidant capacity, limiting its biological action. These antioxidant systems are limited after chronic exposure, reaching damaging levels of free radicals. In this case, it causes oxidative damage, skin lesions, inflammation in the affected area, immunosuppression, premature aging and skin cancer. Therefore, the intervention of exogenous antioxidants is an interesting strategy to limit and inhibit photo-oxidation, providing a photoprotective potential to support the endogenous antioxidant system. This can be achieved through the intervention of exogenous antioxidants, such as carotenoids, phenols, flavonoids and vitamins, expanding options for photoprotective products that could include supplementation with oral, topical and ocular antioxidants [70,71].

Exogenous antioxidants such as carotenoids are fundamental pieces for systemic photoprotection in humans. An exhaustive search indicates that carotenoids such as Astaxanthin, fucoxanthin, β-Carotene and lycopene have acted as sunscreens, reducing the risk of inducing pathologies related to oxidative stress induced by UVR [38,68,69,70]. However, there is not enough scientific information about their properties when applied to the different blood phenotypes, which are not only found in erythrocytes but also in skin cells and other cell lines. Fucoxanthin and β-Carotene have high antioxidant activity, capable of acting as photoprotective agents in human cells. Despite presenting photoinstability, this carotenoid is considered a promising blood group-dependent photoprotective agent since it managed to effectively protect groups A RhD+ve and A RhD-ve (UV-A and UV-B). These results could contribute to generating targeted drugs, nutraceuticals or functional foods for a population highly susceptible to developing conditions related to overexposure to UVR. The results are promising, since group A RhD-ve was the most susceptible to phototoxicity induced by UV-A and UV-B radiation.

In addition to carotenoids, antioxidants such as phenols, flavonoids and vitamins participate in the prevention of skin cancer associated with ultraviolet radiation. Polyphenols such as gallic acid are ideal chemopreventive agents for counteracting skin disorders. Particularly, recent advances suggest that gallic acid can suppress, slow and reverse the process of skin carcinogenesis [35,41,71]. The photoprotective potential of these polyphenols has reduced oxidative stress and DNA damage and suppressed the immune response. Additionally, they can nullify the various biochemical processes mediated by ultraviolet radiation at in vitro and in vivo levels. Polyphenols can act as photoprotective agents since they have the ability to absorb radiation in the length spectrum of UV-A and UV-B radiation. However, this characteristic has only been detected in those polyphenols that are yellow, red or purple. Since gallic acid is a transparent compound, the photoprotective mechanism is probably different. In our study, the photoprotective effect of gallic acid depended on the blood groups tested, where the most protected groups were A RhD+ve (UV-A), A RhD+ve, B RhD+ve and AB RhD-ve (UV-B). Therefore, gallic acid can be used as an inhibitor of ultraviolet radiation-induced oxidative damage in people with type B RhD+ve, since they are the blood type most susceptible to UVR according to the results obtained.

Unlike carotenoids and polyphenols, ascorbic acid (Vitamin C) is not considered a sunscreen due to its chemical nature, because vitamin C does not absorb light in the UV-A or UV-B spectrum. However, vitamin C has a potential antioxidant that is praised for its antiaging benefits. For this reason, vitamin C has been shown in studies to protect against oxidative damage induced by UV radiation by acting as a sunscreen. There are proteins that transport vitamin C to the site of inflammation that has come into contact with ultraviolet light in harmful amounts. As a consequence, vitamin C increases in keratinocytes, resulting in the need to absorb a higher concentration of vitamin C for adequate protection. In cultured keratinocytes exposed to UVR, vitamin C addition reduces oxidative damage to DNA and lipid peroxidation, prevents lysosomal degradation, inhibits the release of pro-inflammatory cytokines and protects against cell death by apoptosis. However, in animal models exposed to UV radiation, the addition of vitamin C to the diet of rodents through oral supplementation reduced the size of skin tumors and the development of dermal neoplastic diseases. However, studies suggest that the combination of vitamin C, vitamin E, RRR-alpha-tocopherol and β-Carotene effectively reduces the damage induced by UV radiation. The synergistic effect would expand the properties of vitamin C as a photoprotective agent in oral supplementation. Our study used erythrocytes as a cellular model for the evaluation of the photoprotective effect of vitamin C on the different blood phenotypes exposed to UV-A and UV-B radiation. The addition of vitamin C inhibited the effects of UV-A in groups A RhD+ve and B RhD+ve, preserving cellular integrity without visible oxidative damage, conferring adequate photoprotection. This effect decreased when erythrocytes were exposed to UV-B light, radiation considered more harmful. In this case, the photoprotective effect decreased in all blood phenotypes. The AB RhD-ve blood group stood out among the others, being the group most protected by vitamin C. The photoprotective potential of vitamin C depended on the surface antigen on the erythrocytes. More studies would have to be carried out in combination with photoprotective antioxidants to evaluate its effect on the groups most susceptible to degradation in the presence of UVR [35,38].

In our research, we explore the hypothesis that the use of antioxidant compounds as preventive treatments can be adapted according to the blood group of patients with chronic pathologies. It is suggested to administer the antioxidant compounds to different groups of healthy volunteers and patients with certain pathologies associated with blood groups to evaluate the effect of adding erythroprotective antioxidants associated with blood groups in the human diet. Subsequently, the bioactive compounds are applied as differential treatments in patients with some chronic pathology such as epilepsy, cancer or diabetes, and their effect is evaluated according to their blood group. The effectiveness of these treatments depends on the in vitro and in vivo studies carried out before the clinical phase and their administration. The possible health implications are explored, and the possibility of using lipid-based nanostructured carriers (nanoliposomes, surfactant-based nanocarriers, nanoemulsions, nanostructured lipid carriers and solid lipid nanoparticles) as effective carriers of erythroprotective antioxidants is raised, since they turn out to be an appropriate pharmacological route, providing greater protection during the digestive process, favoring controlled release. The nanodimensional architecture of lipid-based nanostructured carriers is especially beneficial in medical applications as it allows them to pass through cell membranes more easily due to their nanoscale size. Our results accompanied by nanotechnology could contribute to the development of viable drugs in the pharmaceutical industry, focused on the design of devices, clinical tests, nutraceuticals and functional foods, designed for people with a specific blood type, preventing chronic diseases that affect a group of the most susceptible population. There is evidence that positions carotenoids as cytoprotective agents (action associated with blood groups) capable of inhibiting free radicals.

## 4. Conclusions

All tested antioxidants exhibited potent antioxidant capacity in FRAP and ABTS. The results indicate that the erythroprotective antioxidants are suitable for use at the concentrations tested, since they did not show toxicity in blood biocompatibility tests. Therefore, the integrity of the erythrocyte membrane is not compromised, and antioxidants even confer an erythrocyte effect on the stability of the membrane against hemolysis induced by heat and hypotonicity, factors that could induce the synthesis of free radicals that promote cellular oxidation. The impact of blood groups on the erythroprotective potential of the antioxidants FXN, β-Car, GA, QUE and AA is evident, providing an erythroprotective effect dependent on blood group. Photoprotective effect and antihemolytic activity increased with the presence of antigen A, inhibiting uncontrolled respiratory discharges. These results are very important, since it has been documented that antigen A is associated with breast and skin cancer, pathologies related to uncontrolled respiratory discharges, induced chemically and by photo-oxidation. Erythroprotective antioxidants stabilized the redox state of the erythrocytes, stimulating the inhibition of lipid peroxidation and proteolysis, an action that could prevent chronic-degenerative pathologies associated with prolonged exposure to ultraviolet radiation or free radicals generated by chemical reactions. These compounds could be applied as additives and nutraceuticals or encapsulated to improve their bioaccessibility, highlighting the importance of developing bio-targeted drugs for the groups most susceptible to certain chronic-degenerative pathologies related to cancer. Beyond the impact of blood groups with the erythroprotective potential of different antioxidants, the susceptibility of the eight blood phenotypes to oxidative stress is reported. It was found that group B is the most susceptible to degradation by free radicals induced by ultraviolet radiation, while groups AB RhD-ve and O RhD-ve were the most susceptible to oxidative stress induced by AAPH. These results could help us understand the mechanisms of association between blood groups with susceptibility to certain types of cancer.

## Figures and Tables

**Table 1:** Antioxidant activities of FXN, β-Car, GA, QUE and AA.

	µmol TE/g ± SD		
Compounds	FRAP	ABTS^•+^	DPPH•
FXN	2687.03 ^b^ ± 173.26	2638.53^c^ ± 244.87	303.15 ^c^ ± 42.66
β-Car	2780.87 ^b^ ± 92.23	2371.99 ^c^ ± 26.79	348.12 ^c^ ± 38.49
GA	5970.45 ^a^ ± 230.07	3124.72 ^b^ ± 133.22	614.74 ^b^ ± 99.77
QUE	896.70 ^c^ ± 19.06	2037.62 ^d^ ± 80.62	3980.61 ^a^ ± 222.58
AA	2684.54 ^b^ ± 104.13	3412.22 ^a^ ± 21.94	4190.9 ^a^ ± 155.16

Data are shown as the mean ± SD (standard deviation) of at least three replicates (n ≥ 3). One-way analysis of Pearson correlation coefficient of the results of FRAP ABTS^•+^ and DPPH• tests showed greater variance (ANOVA). Different lowercase letters represent significant differences in the antioxidant activity of each compound (*p* < 0.001). FXN, fucoxanthin; β-Car, β-Carotene; GA, gallic acid; QUE, quercetin; AA, ascorbic acid.

**Table 2:** Antihemolytic activity of fucoxanthin, β-Carotene, gallic acid, quercetin and ascorbic acid against the oxidative hemolysis induced by AAPH on different ABO RhD+ve blood types.

	Inhibition of Hemolysis (%)			
	Different Blood Groups (RhD+ve)			
Compounds	A	B	AB	O
FXN	100.70 ^Aa^ ± 0.71	79.57 ^Bd^ ± 1.13	97.30 ^Bb^ ± 1.37	91.15 ^Bc^ ± 1.04
β-Car	101.05 ^Aa^ ± 3.78	84.82 ^Ac^ ± 2.56	98.87 ^ABb^ ± 0.49	97.05 ^Ab^ ± 0.31
GA	94.42 ^Bb^ ± 1.08	77.16 ^Bd^ ± 0.49	99.43 ^Aa^ ± 1.37	81.94 ^Dc^ ± 1.08
QUE	95.61 ^Bb^ ± 0.41	70.78 ^Cc^ ± 0.64	100.85 ^Aa^ ± 1.43	83.51 ^Cb^ ± 0.61
AA	93.12 ^Bb^ ± 3.55	62.84 ^Dd^ ± 3.08	99.29 ^Aa^ ± 1.07	83.33 ^CDc^ ± 3.26

Data are shown as the mean *±* SD (standard deviation) of at least three replicates (n ≥ 3). One-way analysis of Pearson correlation coefficient of the results of the inhibition of hemolysis (%) tests showed greater variance (ANOVA). Different capital letters represent significant differences via a one-way ANOVA between the antioxidant compounds and each blood group (*p* < 0.001). Different lowercase letters represent significant differences via a one-way ANOVA between the blood groups and each antioxidant compound (*p* < 0.001). FXN, fucoxanthin; β-Car, β-Carotene; GA, gallic acid; QUE, quercetin; AA, ascorbic acid.

**Table 3:** Antihemolytic activity of fucoxanthin, β-Carotene, gallic acid, quercetin and ascorbic acid against the oxidative hemolysis induced by AAPH on different ABO RhD-ve blood types.

	Inhibition of Hemolysis (%)			
	Different Blood Groups (RhD-ve)			
Compounds	A	B	AB	O
FXN	79.93 ^Ab^ ± 3.28	92.41 ^Aa^ ± 1.34	81.28 ^Bb^ ± 1.28	91.15 ^Ba^ ± 1.43
β-Car	73.72 ^Bd^ ± 1.47	90.86 ^Ab^ ± 2.63	78.30 ^Bc^ ± 1.53	97.05 ^Aa^ ± 2.43
GA	75.30 ^Bd^ ± 2.34	89.76 ^Ab^ ± 1.18	95.60 ^Aa^ ± 1.30	81.94 ^Cc^ ± 2.66
QUE	50.85 ^Cc^ ± 2.56	82.44 ^Ba^ ± 2.45	62.55 ^Db^ ± 4.60	83.51 ^Ca^ ± 1.65
AA	80.66 ^Ab^ ± 3.39	91.02 ^Aa^ ± 1.11	74.75 ^Cc^ ± 1.30	83.33 ^Cb^ ± 4.21

Data are shown as the mean *±* SD (standard deviation) of at least three replicates (n ≥ 3). One-way analysis of Pearson correlation coefficient of the results of the inhibition of hemolysis (%) tests showed greater variance (ANOVA). Different capital letters represent significant differences via a one-way ANOVA between the antioxidant compounds and each blood group (*p* < 0.001). Different lowercase letters represent significant differences via a one-way ANOVA between the blood groups and each antioxidant compound (*p* < 0.001). FXN, fucoxanthin; β-Car, β-Carotene; GA, gallic acid; QUE, quercetin; AA, ascorbic acid.

## Data Availability

The original contributions of data presented in this research are included in the article; further inquiries can be directed to the corresponding authors.
